# DNA- and RNA-Binding Proteins Linked Transcriptional Control and Alternative Splicing Together in a Two-Layer Regulatory Network System of Chronic Myeloid Leukemia

**DOI:** 10.3389/fmolb.2022.920492

**Published:** 2022-08-16

**Authors:** Chuhui Wang, Xueqing Zong, Fanjie Wu, Ricky Wai Tak Leung, Yaohua Hu, Jing Qin

**Affiliations:** ^1^ School of Pharmaceutical Sciences (Shenzhen), Sun Yat-sen University, Shenzhen, China; ^2^ College of Professional and Continuing Education, The Hong Kong Polytechnic University, Hong Kong, China; ^3^ Shenzhen Key Laboratory of Advanced Machine Learning and Applications, College of Mathematics and Statistics, Shenzhen University, Shenzhen, China

**Keywords:** DNA- and RNA-binding protein, transcription factor, splicing factor, transcriptional regulatory network, alternative splicing regulatory network, chronic myeloid leukemia

## Abstract

DNA- and RNA-binding proteins (DRBPs) typically possess multiple functions to bind both DNA and RNA and regulate gene expression from more than one level. They are controllers for post-transcriptional processes, such as splicing, polyadenylation, transportation, translation, and degradation of RNA transcripts in eukaryotic organisms, as well as regulators on the transcriptional level. Although DRBPs are reported to play critical roles in various developmental processes and diseases, it is still unclear how they work with DNAs and RNAs simultaneously and regulate genes at the transcriptional and post-transcriptional levels. To investigate the functional mechanism of DRBPs, we collected data from a variety of databases and literature and identified 118 DRBPs, which function as both transcription factors (TFs) and splicing factors (SFs), thus called DRBP-SF. Extensive investigations were conducted on four DRBP-SFs that were highly expressed in chronic myeloid leukemia (CML), heterogeneous nuclear ribonucleoprotein K (HNRNPK), heterogeneous nuclear ribonucleoprotein L (HNRNPL), non-POU domain–containing octamer–binding protein (NONO), and TAR DNA-binding protein 43 (TARDBP). By integrating and analyzing ChIP-seq, CLIP-seq, RNA-seq, and shRNA-seq data in K562 using binding and expression target analysis and Statistical Utility for RBP Functions, we discovered a two-layer regulatory network system centered on these four DRBP-SFs and proposed three possible regulatory models where DRBP-SFs can connect transcriptional and alternative splicing regulatory networks cooperatively in CML. The exploration of the identified DRBP-SFs provides new ideas for studying DRBP and regulatory networks, holding promise for further mechanistic discoveries of the two-layer gene regulatory system that may play critical roles in the occurrence and development of CML.

## Introduction

Nucleic acid–binding proteins (NBPs), including DNA-binding proteins (DBPs) and RNA-binding proteins (RBPs), can regulate genes by interacting with DNAs or RNAs. DBPs and RBPs were considered to be functionally distinctive and were studied independently. However, this is an outdated concept as there is an increasing number of evidence suggesting that there are no well-defined differences between DBPs and RBPs, whereas many proteins are capable of interacting with both nucleic acids. These proteins are called DNA- and RNA-binding proteins (DRBPs) (Hudson and Ortlund 2014; Leung et al., 2019). DRBPs typically possess multiple functions and regulate gene expression from more than one level. They are capable of controlling post-transcriptional processes, such as splicing, polyadenylation, capping, modification, export, localization, translation, turnover, and degradation of RNA transcripts in eukaryotic organisms, as well as transcriptional regulation (Shi et al., 2007; Glisovic et al., 2008; Poon and Chen 2008).

However, the identification of DRBPs is challenging for a few reasons: 1) not a single experimental technique is available for directly identifying DRBPs (Zheng et al., 2016), 2) current databases do not contain information on high-confidence DRBPs (Yan and Kurgan 2017), 3) DRBPs cannot be perfectly predicted from domain structures (Yan et al., 2016), 4) the existing literature are highly heterogeneous concerning DRBPs (Yan and Kurgan 2017), and finally, 5) the electronic annotations for DRBPs are nonuniform (Zhang J. et al., 2020). It is fortunate that the experimental methods to identify DBPs and RBPs globally are available. For example, chromatin immunoprecipitation with high-throughput sequencing (ChIP-seq) is a widely used approach to reveal protein–DNA interactions *in vivo* (Schmidt et al., 2009). Ultraviolet crosslinking and immunoprecipitation coupled with next-generation sequencing (CLIP-seq) is the most important means for determining the binding sites of RBPs on a transcriptome-wide level (Uhl et al., 2017). The strategies we adopted here are cross-comparison of the public DBP and RBP datasets and identification of the intersected members, such as DRBPs.

Several popular high-quality DBP and RBP databases are available online and extensively used. For example, CIS-BP is an online library of transcription factors (TFs) and their DNA-binding motifs (Weirauch et al., 2014). AnimalTFDB provides resources with the most comprehensive and accurate information on animal TFs and cofactors (Hu et al., 2019). “The Human Transcription Factors” contains the official list of human TFs that were manually examined by a panel of experts based on available data (Lambert et al., 2018). RBPbase (https://rbpbase.shiny.embl.de/) integrates datasets from high-throughput RNA–binding protein (RBP) detection studies. RNA-binding proteins database (RBPDB) (Cook et al., 2011) is a database focusing on the collection of experimentally validated RBPs and RNA-binding domains. CISBP-RNA (Ray et al., 2013) and ATtRACT (Giudice et al., 2016) are also online libraries of RBPs. DRNApred, a server that provides prediction of DNA- and RNA-binding residues, provides annotated DRBP datasets (Yan and Kurgan 2017).

**FIGURE 1:**
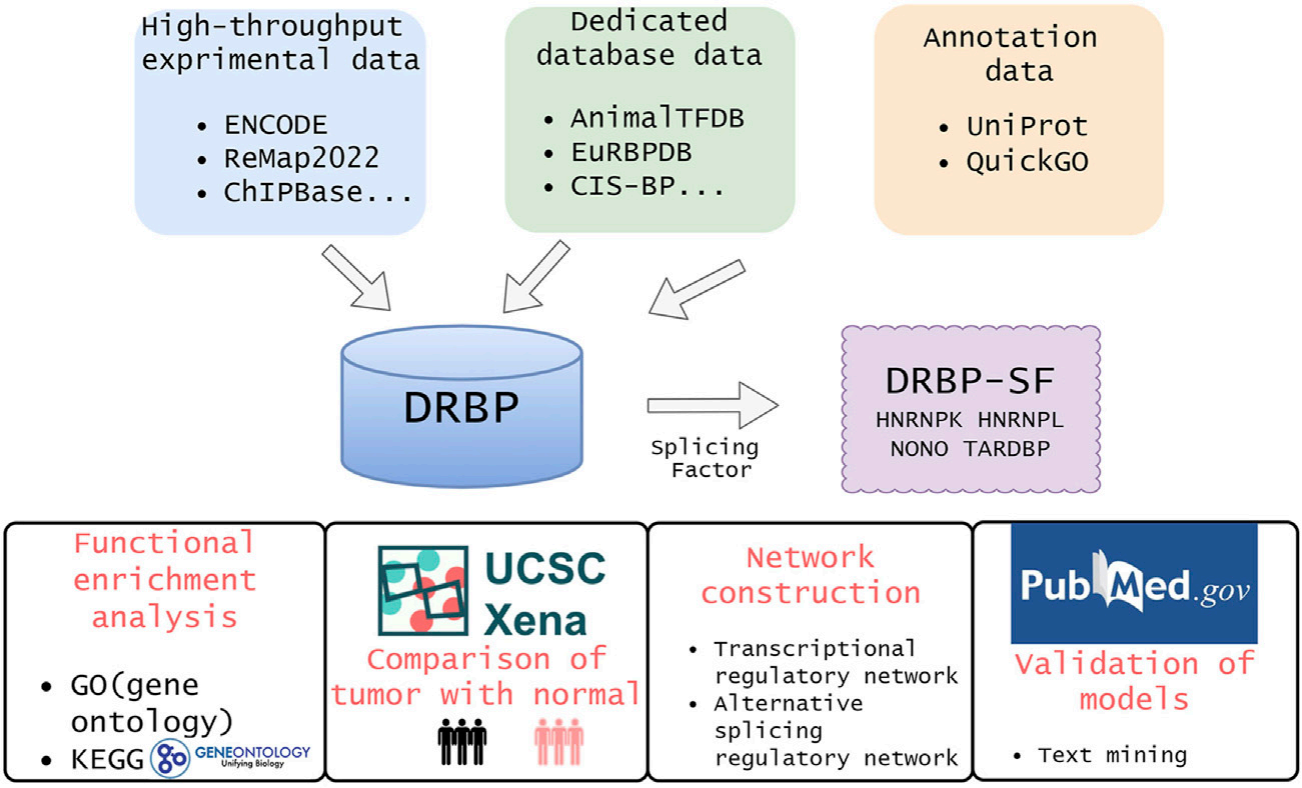
Overview of this study. We collected DRBPs from high-throughput, dedicated database, and annotation data. Then, the intersection of DRBP and splicing factor lists are defined as DRBP-SFs. To investigate the functions of DRBP-SFs, we carried out differential expression analysis, functional enrichment analysis and network construction for HNRNPK, HNRNPL, NONO and TARDBP in chronic myeloid leukemia. At last, we verified the model by text mining.

Although DRBPs are reported to play critical roles in various developmental processes and diseases, it is still unclear how they work with DNAs and RNAs simultaneously and regulate genes at both transcriptional and post-transcriptional levels. To tackle this question, we collected data from a variety of databases and literature mentioned above and identified DRBPs as well as investigated the functional mechanism of DRBPs (Figure 1). Functional enrichment analysis revealed that DRBPs are enriched with splicing factors (SFs), suggesting that proteins called DRBP-SFs can link transcriptional and alternative splicing (AS) regulatory networks together. Previous studies have paid attention to the regulatory network of TFs and SFs at a single regulatory level (Qin et al., 2011; Ule and Blencowe 2019; Takaku et al., 2020). However, the occurrence and development of cancer often result from the dysregulation of multiple layers of gene regulatory networks. For instance, disturbance of a controlled epithelial balance during cancer progression is triggered by altering several layers of gene regulation, including transcriptional and translational machinery, expression of noncoding RNAs, AS, and protein stability (De Craene and Berx 2013). Comprehensive knowledge of factors that regulate these networks is lacking. Therefore, it is necessary to investigate the underlying regulatory mechanism by constructing multilayer networks from the unique perspective of the multifunctionality of DRBP-SFs. Binding and expression target analysis (BETA) is a software package that integrates ChIP-seq or chromatin regulators with differential expression data to infer direct target genes of TFs (Wang et al., 2013). SURF, Statistical Utility for RBP Functions, is a new integrative framework for the analysis of large-scale CLIP-seq and coupled RNA-seq data from ENCODE consortium data (Chen and Keles 2020). By integrating and analyzing the ChIP-seq, CLIP-seq, RNA-seq, and shRNA-seq data of K562 using BETA and SURF, we constructed a two-layer regulatory network system associated with DRBP-SFs in chronic myeloid leukemia (CML). Based on the two-layer network, we proposed three regulatory modes of how DRBP-SFs connect transcriptional and AS regulatory networks cooperatively. Emerging solid evidence showed that TFs and SFs rarely function alone, in general, and they all need to cooperate with other factors (Feng et al., 2020). Hence, it is worth probing which factors they cooperate with and whether there is a regulatory relationship between these cooperative partners. Our proposed models II and III may provide some evidence. This study provided a novel DRBP multitasking paradigm with supporting evidence, where DRBPs were demonstrated to co-regulate DNA and RNA in conjunction.

## Materials and Methods

### Search Strategy for DNA- and RNA-Binding Protein and DNA- and RNA-Binding Protein Reliability Ranking

As of September 2021, we collected 995 DRBPs (Supplementary Table S1) from three sources: 1) high-throughput data, 2) dedicated database data, and 3) annotation data (Table 1). NBPs documented as DBPs as well as RBPs from various sources were identified as DRBPs here. The reliability of DRBP collected from different sources is ranked from high to low as follows: DRBPs with high-throughput nucleic acid–binding data, DRBPs from databases or literatures with experimental data support, DRBPs annotated as DBP and RBP, and proteins predicted as DBP and RBP. In general, the accuracy of DBP or RBP assertions would be higher if the evidence were derived from experiments. The credibility of the protein was evaluated from two aspects, which are the evidence of DNA binding and RNA binding. The aspect with lower credibility was regarded as the credibility of the protein (Table 1).

**TABLE 1:** Data sources for DBPs and RBPs.

Data source	Data type	Database	Features	Reference	Confidence of proteins only appearing in this database
High-throughput data	ChIP-seq	ReMap2022	ReMap is a large-scale integrative analysis of DNA-binding experiments for *Homo sapiens*, *Mus musculus*, *Drosophila melanogaster*, and *Arabidopsis thaliana* transcriptional regulators.	Hammal et al. (2022)	High
		ChIPBase	ChIPBase, an integrated resource and platform for decoding transcription factor binding maps, expression profiles, and transcriptional regulation of long noncoding RNAs (lncRNAs, lincRNAs), microRNAs, other ncRNAs (snoRNAs, tRNAs, snRNAs, etc.), and protein-coding genes from the ChIP-seq data.	Zhou et al. (2017)	High
		JASPAR	JASPAR is an open-access database of curated, nonredundant transcription factor (TF) binding profiles stored as position frequency matrices and TF flexible models for TFs across multiple species in six taxonomic groups.	Castro-Mondragon et al. (2022)	High
		ENCODE	The ENCODE Consortium is an international collaboration of research groups funded by the National Human Genome Research Institute.	Dunham et al. (2012)	High
	CLIP-seq	ENCODE	The goal of ENCODE is to build a comprehensive parts list of functional elements in the human genome, including elements that act at the protein and RNA levels, as well as regulatory elements that control cells and circumstances in which a gene is active.	Dunham et al. (2012)	high
		StarBase	StarBase is designed for decoding Pan-Cancer and Interaction Networks of lncRNAs, miRNAs, competing endogenous RNAs (ceRNAs), RNA-binding proteins (RBPs), and mRNAs from large-scale CLIP-seq (HITS-CLIP,PAR-CLIP, iCLIP, CLASH) data and tumor samples.	Yang et al. (2011)	High
		CLIPdb	CLIPdb is a CLIP-seq database for protein–RNA interactions and aims to characterize the regulatory networks between RBPs and various RNA transcript classes by integrating large amounts of CLIP-seq (including HITS-CLIP, PAR-CLIP, and iCLIP as variations) datasets.	Yang et al. (2015)	High
Dedicated database data	DBP	POSTAR	POSTAR is one of the largest and first integrative resources and platforms incorporating various post-transcriptional regulation events. It enables the experimental biologists to connect protein–RNA interactions with multilayer information of post-transcriptional regulation and functional genes and helps them generate novel hypotheses about the postregulatory mechanisms of phenotypes and diseases.	Hu et al. (2017)	High
		The Human Transcription Factors	The “HumanTFs” website displays the 1,639 known or likely human TFs, with a separate page for each TF, along with all known motifs and information and sequence alignments for each dielectric barrier discharge type.	Lambert et al. (2018)	High
		CIS-BP	CIS-BP is an online library of transcription factors and their DNA-binding motifs.	Weirauch et al. (2014)	High
		AnimalTFDB	The Animal Transcription Factor DataBase (AnimalTFDB) is a resource aimed at providing the most comprehensive and accurate information for animal TFs and cofactors.	Hu et al. (2019)	Medium
		CISBP-RNA	CISBP-RNA is the online library of RNA-binding proteins and their motifs.	Ray et al. (2013)	High
	RBP	RBPbase	RBPbase is a database that integrates high-throughput RBP detection studies.	https://rbpbase.shiny.embl.de/	High
		RNA-binding proteins database (RBPDB)	RBPDB is a collection of experimental observations of RNA-binding sites.	Cook et al. (2011)	High
		ATtRACT	ATtRACT compiles information on 370 RBPs and 1583 RBP consensus–binding motifs, 192 of which are not present in any other database.	Giudice et al. (2016)	High
		EuRBPDB	EuRBPDB is a comprehensive and user-friendly database for eukaryotic RBPs. It contains 315,222 RBPs (forms 6,368 ortholog groups) from 162 eukaryotic species, including human, mouse, fly, worm, and yeast.	Liao et al. (2020)	Medium
Annotation data	DBP and RBP	QuickGO	QuickGO is a web-based tool that allows easy browsing of the Gene Ontology (GO) and all associated electronic and manual GO annotations provided by the GO Consortium annotation groups.	Binns et al. (2009)	Medium
		UniProt	The aim of the UniProt Knowledgebase is to provide users with a comprehensive, high-quality, and freely accessible set of protein sequences annotated with functional information.	Bateman et al. (2019)	Low

### Identification of Splicing Factors in the DNA- and RNA-Binding Protein Set

We collected a total of 545 SFs (Supplementary Table S1) from the following two sources: 1) databases: a. 71 SFs listed in the human SF database SpliceAid-F (Giulietti et al., 2013), b. 323 genes annotated as splicing-related genes in the protein database UniProt (Bateman, et al., 2019); 2) literatures: a total of 479 SFs that have been confirmed by literatures or experiments compiled by other researchers (Sebestyen et al., 2016; Seiler et al., 2018; Zhang D. et al., 2020). The SF and DRBP datasets were cross-compared, and a total of 118 proteins were found to be shared by both datasets. We named them DRBP-SFs here (Supplementary Table S1).

### Bioinformatics Analysis on DNA- and RNA-Binding Proteins

Gene Ontology (GO) annotation enrichment test was used to explore the functional roles of DRBPs in terms of biological process (BP), cellular component (CC), and molecular function (MF). Kyoto Encyclopedia of Genes and Genomes (KEGG) analysis was conducted to search metabolic pathways that DRBPs involved. GO and KEGG analyses were performed using the R package clusterProfiler v4.2.1 (Wu et al., 2021). GO terms and KEGG pathways with *p*-value < 0.01 were considered significantly enriched with DRBPs. Venn diagram was plotted to show the number of overlapping genes using the jvenn tool (Bardou et al., 2014).

### Analysis on Gene Expression Differences Between Chronic Myeloid Leukemia Cells and Whole Blood Normal Cells

RNA-seq data of 70 CML and 337 whole blood normal samples were downloaded from GTEx *via* UCSC Xena (Goldman et al., 2020). Quantile normalization and estimation of mean–variance relationships for log counts were performed using the voom method (Law et al., 2014). Linear model fitting, empirical Bayesian analysis, and differential expression analysis were then performed using limma v3.50.0 (Ritchie et al., 2015). Genes were considered differentially expressed if the absolute value of log 2-fold change was >1 with the adjusted *p*-value < 0.01. We used GEPIA (Gene Expression Profiling Interactive Analysis) to analyze gene correlation for differentially expressed genes and performed principal component analysis dimensionality reduction on two datasets called “Cells-Leukemia cell line (CML)” and “Whole Blood” (Tang et al., 2017).

### DNA- and RNA-Binding Protein Transcriptional and Splicing Regulatory Network Construction

BETA combined the information of binding site and differential expression to score the regulatory potential of each target gene and infer the target genes. To construct transcription network the ChIP-seq data of four DRBP-SFs in K562 cell line was downloaded from ENCODE database. The four DRBP-SFs are heterogeneous nuclear ribonucleoprotein K (HNRNPK), heterogeneous nuclear ribonucleoprotein L (HNRNPL), non-POU domain-containing octamer-binding protein (NONO), and TAR DNA-binding protein 43 (TARDBP). And the IDs of their ChIP-seq data are ENCFF505RNR, ENCFF854WAP, NCFF211TTD and ENCFF564QOL, respectively. The information of differential expression was the CML differential expression files obtained in the previous step. The four ChIP-seq data were successively input into BETA V1.0.7, and the target genes of each DRBP-SF were inferred in combination with the differential expression data. We used the default parameters, except that the threshold of the Kolmogorov–Smirnov test was set to 0.05.

To have an integral understanding of the specific roles of these four DRBP-SFs in AS, we selected four AS events in the SURF database, exon skipping (ES), alternative 3′ (A3SS) or 5′ (A5SS) splicing, and intron retention (RI) to extract splicing regulatory networks. The results of BETA and SURF were imported into Cytoscape v3.9.0 (Shannon et al., 2003) to visualize the two-layer regulatory networks connecting transcriptional regulation and alternative splicing regulation through DRBP-SFs.

### Protein–Protein Interaction Network Analysis and Gene–Disease Association Analysis

To identify co-regulators of DRBP-SF, we searched protein–protein interactions (PPIs) between DRBP-SFs and TFs/SFs in the String database (Szklarczyk et al., 2021). We imported SF regulated by DRBP-SF at the transcriptional level and DRBP-SF itself or TF regulated by DRBP-SF at the splicing level and DRBP itself into the String database, extracted interactions among them with the confidence score >0.7 or 0.4, and generated the PPI network with the meaning of network edges as confidence. Edges between DRBP-SFs and SFs/TFs in PPI networks represent protein–protein associations. To explore the biological significance of the proposed regulatory model, we performed a disease association analysis of the co-regulated genes of the three models of HNRNPK using DisGeNET (Pinero et al., 2020), a knowledge platform for disease genomics.

### Heterogeneous Nuclear Ribonucleoprotein K Binding Sequence Motif Scanning

Motif analysis was performed using MEME Suite 5.4.1 (Bailey et al., 2015). For DNA binding, peaks from ChIP-seq data (HNRNPK: ENCSR014RCS) were adjusted to 500 bp in length, followed by DNA sequence extraction using Bedtools getfasta (Quinlan and Hall 2010). MEME-chIP was utilized for motif scanning. The *in vitro* DNA-binding motif of HNRNPK has not been experimentally validated; thus, the top 3 *de novo* motifs with the smallest *p*-value from MEME (Bailey and Elkan 1994) or STREME (Bailey, 2021) were regarded as HNRNPK DNA-binding motifs. They were then subsequently inputted for scanning peak regions with proximity to target genes by find individual motif occurrences (FIMO) (Grant et al., 2011). For transcriptional regulations, peak regions were defined as the “associate_peaks” output by BETA, that is, the peaks within 100 kb from the transcription starting site of each gene. When a HNRNPK motif was found within the peak region of a target gene, the promoter region of this gene is considered to be directly bound by HNRNPK.

For RNA binding, peaks from enhanced CLIP (eCLIP) data (HNRNPK: ENCSR268ETU) were adjusted to 100 bp. Bedtools getfasta was used to convert the peak coordinates into RNA sequence by taking strand information into consideration. Then, MEME-chIP was used for motif discovery. RNA-binding motifs discovered by MEME-chIP were compared with motifs of HNRNPK downloaded from the database CISBP-RNA by Tomtom (Gupta et al., 2007), with a *q*-value < 0.05. It was regarded as a true direct-binding motif when the *in vivo* RNA-binding motif derived from eCLIP data matched an *in vitro* RNA-binding motif from the CISBP-RNA database (Ray et al., 2013). CISBP-RNA is an online library of RBPs and their motifs derived from RNAcompete experimental techniques, so we consider the motifs from this database to be high-confidence direct-binding motifs. Then, the HNRNPK RNA-binding motifs were utilized to scan the peak regions of the target genes by FIMO. For RNA splicing regulations, a gene was defined as a target gene when RBP binding signals were captured in any position of the gene region using Bedtools. When a RBP motif was detected in the peak region located in the target gene, HNRNPK was considered to be directly interacting with the target gene’s pre-RNA.

## Results

### Collection of DNA- and RNA-Binding Proteins and Splicing Factors

**FIGURE 2:**
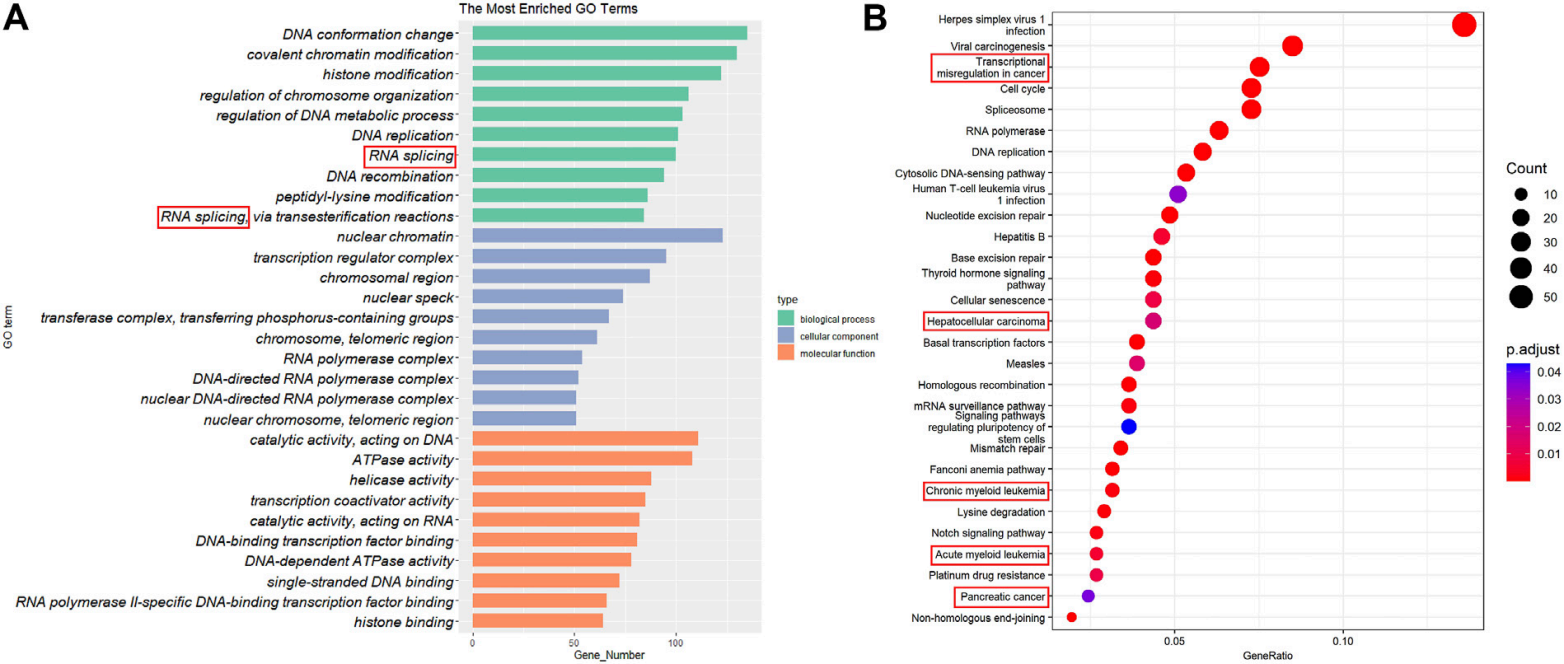
Gene Ontology (GO) annotation and Kyoto Encyclopedia of Genes and Genomes (KEGG) pathway enrichment analysis. **(A)** The top 20 GO terms in biological process, cellular components, and molecular functions. **(B)** The top 29 KEGG pathways.

From various resources listed in Table 1, 995 proteins that possess both DNA- and RNA-binding capabilities were collected as DRBPs (Supplementary Table S1). Functional enrichment analysis was performed for all the DRBPs, and the top 10 terms with their enriched gene counts are presented in Figure 2A. As expected, most of these terms are related to DNA binding and transcriptional regulation. Besides, RNA splicing is also enriched. It suggests that these genes have functions of both DBPs and RBPs, and they might regulate transcription and AS together (Hudson and Ortlund 2014). Indeed, 118 DRBPs were also known as SFs that are functional in RNA splicing; thus, in this study, we classified them as DRBP-SFs (Supplementary Table S1) and investigated their functions in connecting transcriptional and splicing regulatory networks.

In addition, KEGG analysis revealed that DRBPs are enriched in many cancer-related pathways (Figure 2B), including transcriptional misregulation in cancer, hepatocellular carcinoma, CML, acute myeloid leukemia, and pancreatic cancer. This is consistent with previous studies that reported dysregulation of various RBPs and DBPs in different cancers (Andersson et al., 2008; Olivier et al., 2010; Lyko 2018; Wang et al., 2019; Shiroma et al., 2020). Given the potential roles of DRBPs in cancers, in the following sections, we focus on exploring the network regulation mechanisms of DRBP-SF in CML.

### Network Construction for Heterogeneous Nuclear Ribonucleoprotein K, Heterogeneous Nuclear Ribonucleoprotein L, Non-POU Domain–Containing Octamer–Binding Protein, and TAR DNA-Binding Protein

To clarify the relationship between DRBP-SFs and cancer, RNA-seq data from CML patients and healthy donors were compared. The results showed that approximately 73% (86 out of 118) of DRBP-SFs were significantly differentially expressed (Supplementary Table S2). Some of them, such as HNRNPK, were reported to be potential diagnostic markers and therapeutic target of CML (Du et al., 2010). This also validated our previous point: DRBP-SFs play roles in cancer progression. To reveal their possible regulatory mechanism, we constructed the transcriptional and splicing regulatory networks in CML. HNRNPK, HNRNPL, NONO, and TARDBP were chosen for further revelation, considering data availability and their significant upregulations in CML.

**FIGURE 3:**
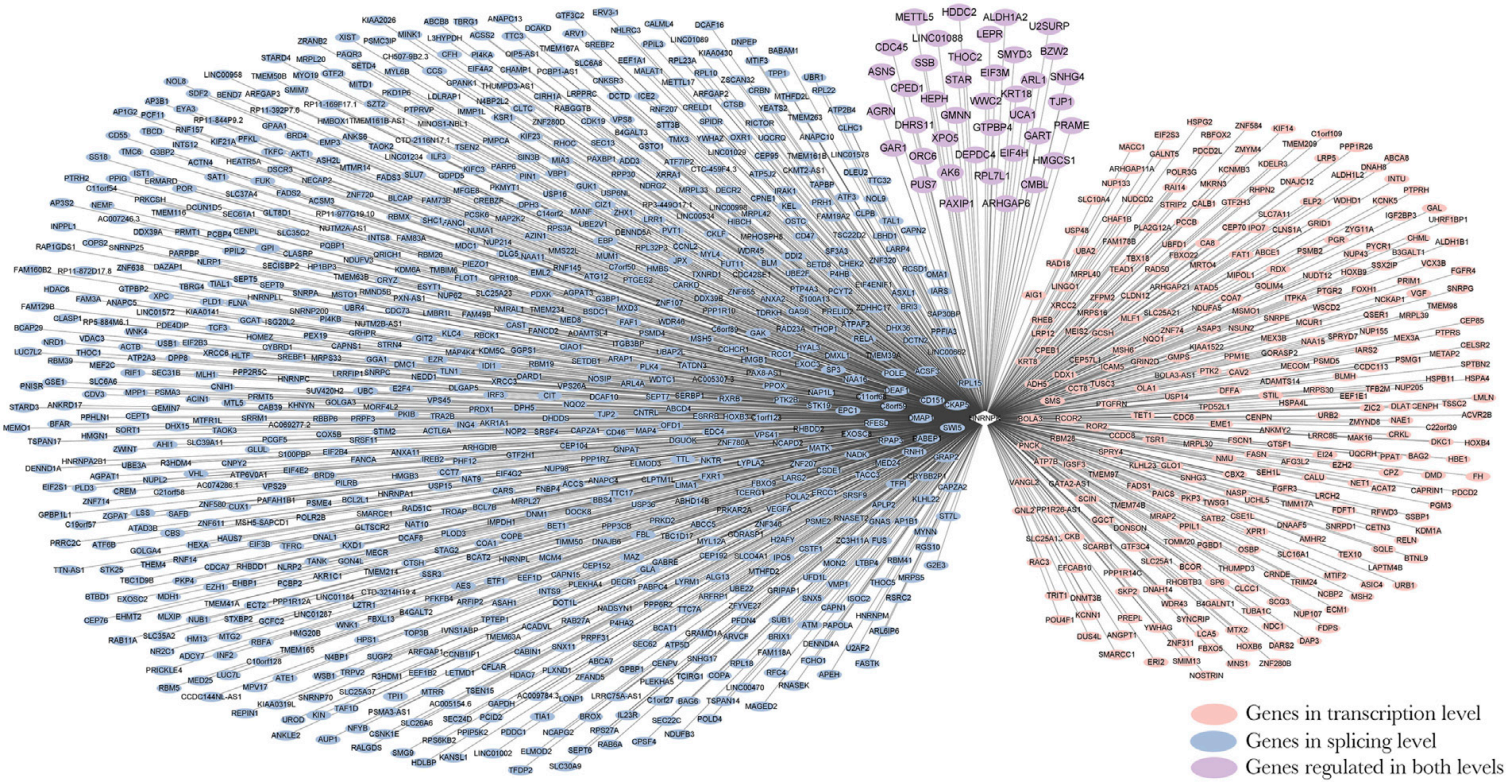
Two-layer regulatory network of Heterogeneous nuclear ribonucleoprotein K (HNRNPK). Pink indicates the target genes of the transcriptional regulatory network of HNRNPK, blue indicates the target genes of splicing regulatory network of HNRNPK, and purple indicates the co-regulated targets of HNRNPK. For more detailed information on genes in the network, please refer to Supplementary Table S7.

**FIGURE 4:**
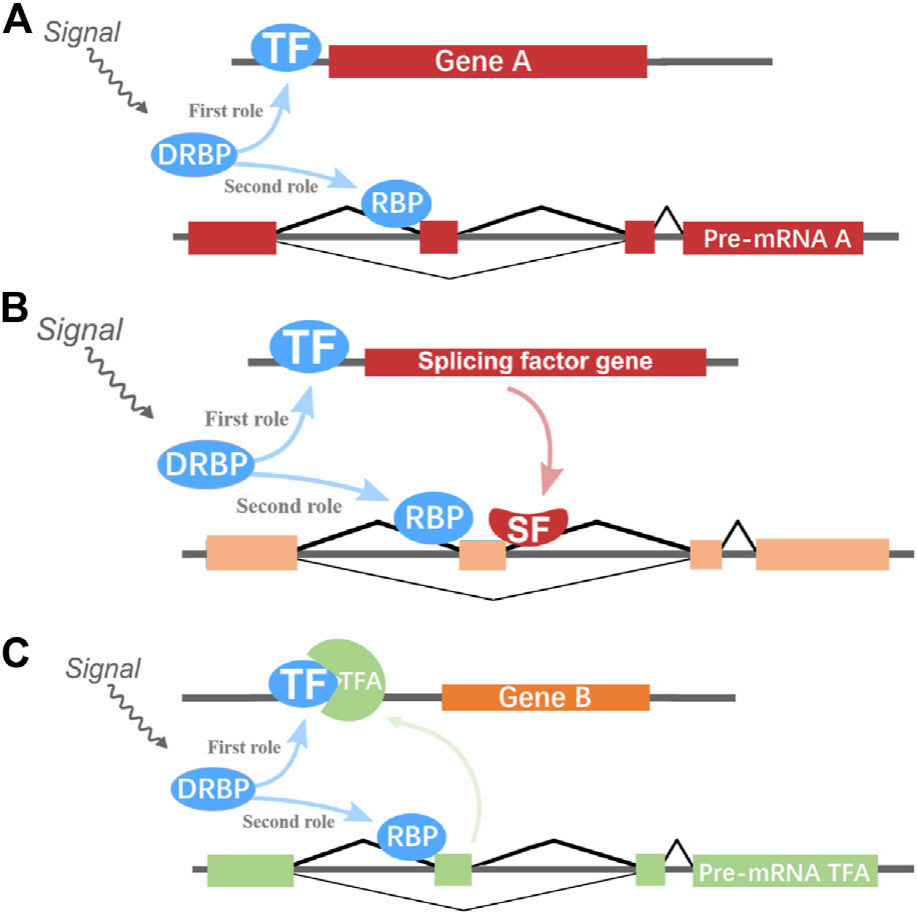
Hypothetical two-layer network regulatory models of genes. **(A)** DNA- and RNA-binding proteins splicing factors (DRBP-SFs) may regulate the same genes at the transcriptional and splicing level as transcription factors (TFs) and SFs, respectively. **(B)** One DRBP-SF may act as SF to regulate the splicing of one gene with another SF controlled by this DRBP-SF in the transcriptional regulation level. **(C)** One DRBP-SF may act as a TF to regulate the transcription of one gene with another TF controlled by this DRBP-SF in the splicing regulation.

By integrating and analyzing the ChIP-seq, CLIP-seq, RNA-seq, and shRNA-seq data using BETA and SURF, we constructed a two-layer regulatory network system controlled by HNRNPK, HNRNPL, NONO, and TARDBP (Figure 3; Supplementary Figures S1–S3). In the two-layer regulatory network system, the four DRBP-SFs were found to connect transcriptional and splicing regulatory networks by regulating target genes at the transcriptional and splicing levels in three different models: I) regulate the same target genes by binding to both their promoters and pre-RNAs concurrently, thereby regulating transcription and splicing simultaneously; II) part of the target genes in the transcriptional regulatory network of the four DRBP-SFs is also SFs that regulate the same target genes in their own splicing regulatory network; while III) part of the target genes in the splicing regulatory network of the four DRBP-SFs is also TFs that regulate the same target genes in their own transcriptional regulatory network (Figure 4).

HNRNPK, HNRNPL, and TARDBP could regulate their own splicing, as well as the splicing activities of each other. For example, HNRNPK can regulate the splicing of HNRNPL; HNRNPL can regulate the splicing of HNRNPK; NONO can regulate the splicing of TARDBP and HNRNPK; while TARDBP can regulate the splicing of HNRNPL and HNRNPK. Besides, the expression of these four genes in CML had a high correlation in a GEPIA analysis (Tang et al., 2017) (Supplementary Figure S4).

### Regulatory Model I

**FIGURE 5:**
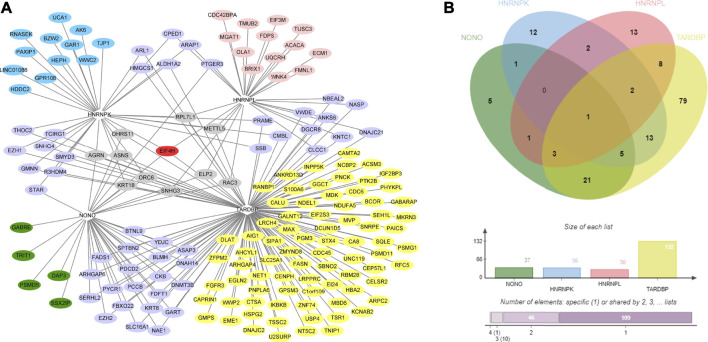
Co-regulatory gene network diagram of the 4 DNA- and RNA-binding proteins splicing factors (DRBP-SFs) and analysis of their Venn analysis. **(A)** Two-layer co-regulated gene network diagram of the four proteins; red indicates a gene co-regulated by all four proteins, gray indicates the genes co-regulated by three proteins, purple indicates the genes co-regulated by two proteins, blue indicates the genes regulated by heterogeneous nuclear ribonucleoprotein K, pink indicates the genes regulated by heterogeneous nuclear ribonucleoprotein L, green indicates the genes regulated by non-POU domain–containing octamer–binding protein, and yellow indicates the genes regulated by TAR DNA-binding protein 43. **(B)** Venn analysis diagram of the co-regulation of the four proteins.

DRBP-SFs may regulate the same genes directly over the transcriptional and splicing levels as TFs and SFs, respectively (Figure 4A). The number of target genes regulated by the four DRBP-SFs by the two different regulatory networks are shown in Table 2, supporting regulation model I. It is worth noting that there were some overlapping genes between the four two-layer gene regulatory systems (Figure 5; Table 2).

**TABLE 2:** Target gene number of two-layer regulatory networks associated with HNRNPK, HNRNPL, NONO, and TARDBP.

Gene symbol	The number of target genes in transcriptional regulatory network	The number of target genes in splicing regulatory network	The number of events in splicing regulatory network	The number of the same target genes in two networks
HNRNPK	668	796	1,057	36
HNRNPL	587	775	1,035	30
NONO	304	760	1,072	37
TARDBP	639	2,197	3,863	132

**FIGURE 6:**
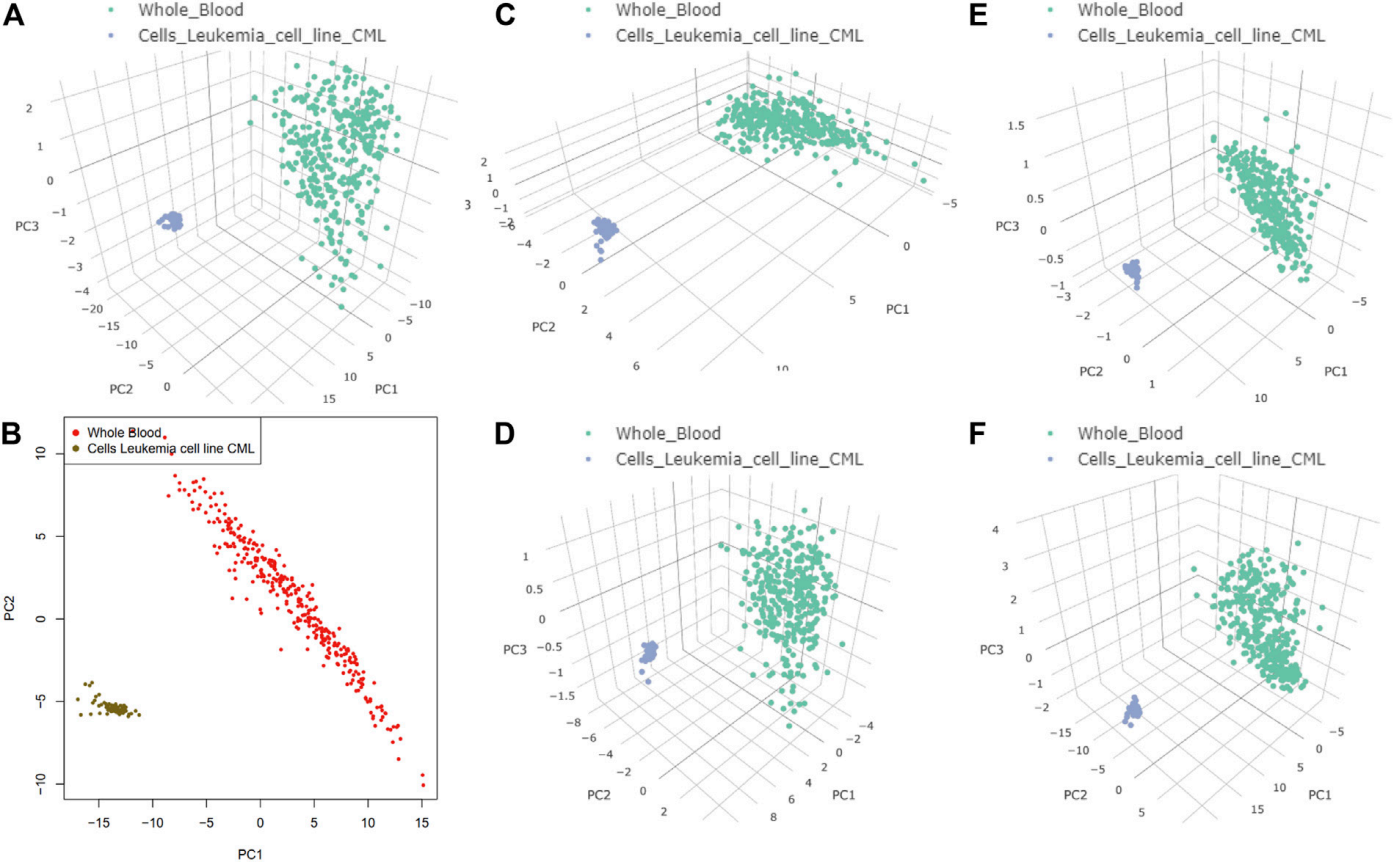
Target genes of the two-layer regulatory networks associated with heterogeneous nuclear ribonucleoprotein K (HNRNPK), heterogeneous nuclear ribonucleoprotein L (HNRNPL), non-POU domain–containing octamer–binding protein, and TAR DNA-binding protein 43 (TARDBP). Principal component analysis dimensionality reduction was performed on the expression datasets of Cells-Leukemia cell line (CML) and Whole Blood. **(A)** Target genes of the two-layer regulatory networks associated with HNRNPK, HNRNPL, NONO, and TARDBP in 3D. **(B)** Target genes of the two-layer regulatory networks associated with HNRNPK, HNRNPL, NONO, and TARDBP in 2D. **(C)** Target genes of the two-layer regulatory networks associated with HNRNPK. **(D)** Target genes of the two-layer regulatory networks associated with HNRNPL. **(E)** Target genes of the two-layer regulatory networks associated with NONO. **(F)** Target genes of the two-layer regulatory networks associated with TARDBP.

The results of dimensionality reduction analysis of the two-level co-regulated genes in CML and whole blood sample are shown in Figure 6, indicating that these co-regulated genes discovered in the process of network construction were closely related to CML but not in whole blood sample. In differential gene analysis, the expression levels of these genes in CML were significantly alternated (Supplementary Table S3). We hypothesized that the co-regulation example we found here was a case of co-transcriptional splicing. Co-transcriptional splicing often occurs in the process of fast transcription and translation (Naftelberg, Schor et al., 2015). Furthermore, the co-regulated genes are highly expressed in CML, which is consistent with the phenomenon of co-transcriptional splicing.

To further investigate the binding modes of DRBP-SF on their targets, we identified both DNA- and RNA-binding motifs for HNRNPK by MEME-chIP. Among the 138 HNRNPK binding motifs enriched in the ChIP-seq peak regions of HNRNPK, we identified 3 *de novo* DNA motifs with the smallest *p*-value as the most likely DNA-binding motifs of HNRNPK (Supplementary Figure S5A). By scanning the DNA-binding ChIP-seq peak regions of HNRNPK with the three motifs, we found that all the 35 genes regulated by model I, except urothelial cancer associated 1 (UCA1), had one of these three motifs in their ChIP-seq peak regions (Supplementary Table S4), which implies direct bindings of HNRNPK at their promoters. After scanning the ChIP-seq peak regions of UCA1 associated with other enriched motifs obtained by MEME-ChIP, a transcription factor E2-alpha (TFE2) motif obtained the smallest *p*-value. Therefore, we inferred that an indirect binding of HNRNPK to UCA1 promoter DNA might be realized by the help of a TF TFE2 binding at this site, which has the MF of cis-regulatory region sequence-specific DNA-binding and has the biological function of positive regulation of DNA-binding TF activity (Kim et al., 2004; Yoon et al., 2011).

From the eCLIP peak regions of HNRNPK, we obtained 87 enriched RNA-binding motifs of HNRNPK by MEME-chIP. There were nine *de novo* motifs discovered, among which three were from MEME and six were from STREME, and the rest were already existing motifs. After comparing the similarity of 9 de novo motifs with a known HNRNPK RNA-binding motif-CCAWMCC (Ray et al., 2013), we selected the three most similar motifs with *q*-value < 0.05 as the RNA-binding motif of HNRNPK (Supplementary Figures S5B, S6). By scanning the HNRNPK eCLIP peak regions with these motifs, we found that 6 out of the 36 genes listed of model I possess HNRNPK binding sites, namely, aldehyde dehydrogenase family 1 (ALDH1A2), ArfGAP with RhoGAP domain, ankyrin repeat and PH domain 1 (ARAP1), cadherin-like and PC-esterase domain–containing 1 (CPED1), dehydrogenase–reductase 11 (DHRS11), 3-hydroxy-3-methylglutaryl-CoA synthase 1 (HMGCS1), and SET and MYND domain–containing 3 (SMYD3). Therefore, HNRNPK is assumed to bind on the pre-mRNA of these six genes directly, whereas the pre-mRNAs of other two-level co-regulated genes of model I may be indirectly bonded to HNRNPK through other SFs. For example, among the other 30 genes, 12 genes possess motif of another SF, serine–arginine-rich splicing factor 2 (SRSF2), which is indispensable for the splicing of pre-mRNA and required for the formation of the earliest ATP-dependent splicing complex and interacts with spliceosomal components bound to both the 5′- and 3′-splice sites during spliceosome assembly (Jang et al., 2009; Edmond et al., 2011).

### Regulatory Model II

A DRBP-SF may act as SF in conjunction with another SF regulated by this DRBP-SF at the transcriptional level during splicing regulation (Figure 4B). For instance, 7.5%–40% of the direct splicing targets of HNRNPK, HNRNPL, NONO, and TARDBP were also regulated by other SFs, such as nuclear cap–binding protein subunit 2 (NCBP2), which is a component of the cap-binding complex (CBC), binding co-transcriptionally to the 5′ cap of pre-mRNAs and involved in pre-mRNA splicing and small RNA-binding exonuclease protection factor La (SSB), which binds to the 3′ poly(U) terminus of nascent RNA polymerase III transcripts (Chambers et al., 1988; Gottlieb and Steitz 1989; Ishigaki et al., 2001; Ray and Das 2002). In contrast, NCBP2 and SSB were also regulated by the four DRBPs at the transcriptional level. NCBP2 and SSB were found to interact with HNRNPK, HNRNPL, NONO, and TARDBP by analyzing the PPI network between target genes with SF/TF functions and the four DRBP-TFs (Supplementary Figures S8–S12).

**FIGURE 7:**
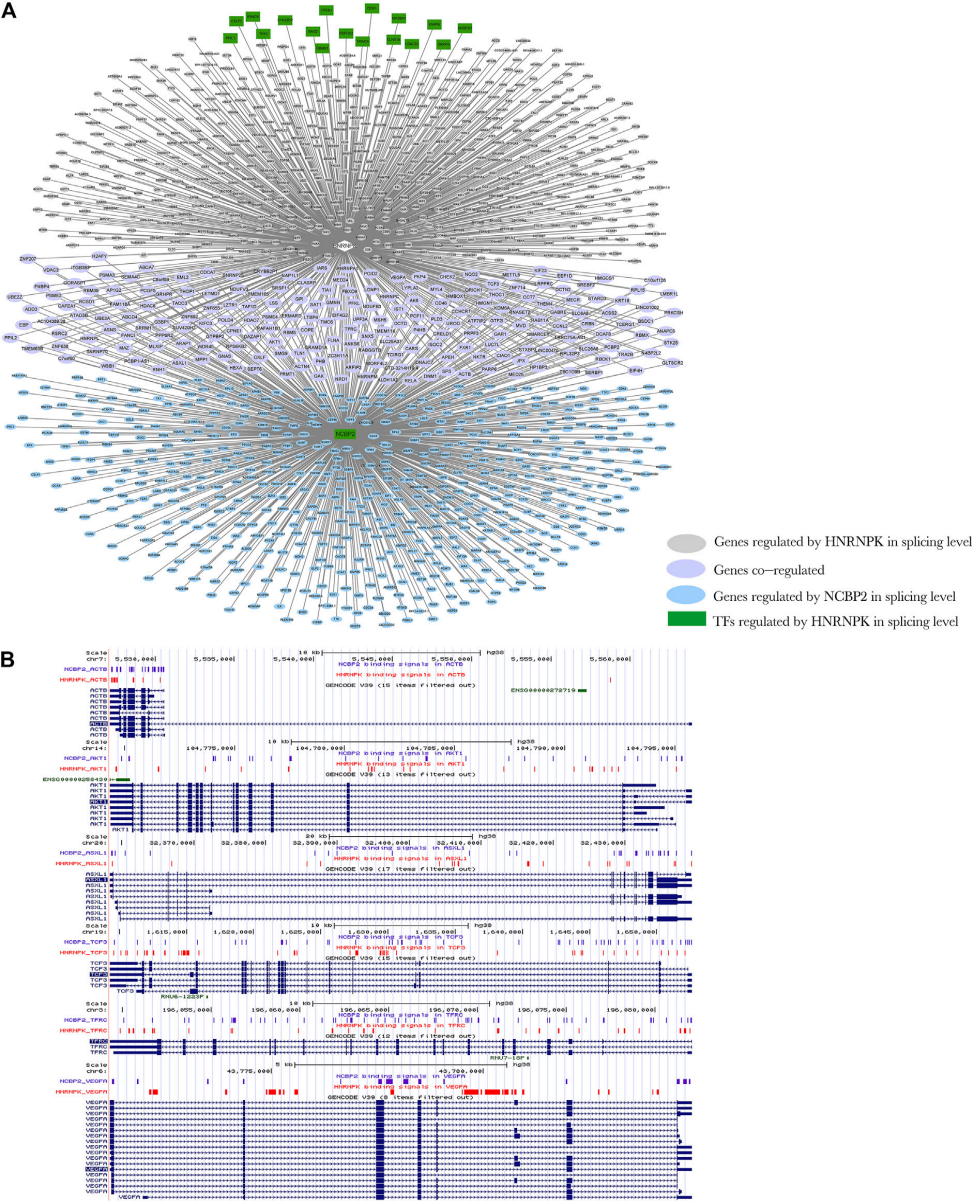
Network diagram of regulation model Ⅱ. **(A)** Heterogeneous nuclear ribonucleoprotein K (HNRNPK) regulates the transcription of nuclear cap–binding protein subunit 2 (NCBP2), and then HNRNPK and NCBP2 can jointly regulate the splicing of genes. Green indicates the genes that act as splicing factors (SFs) regulated by HNRNPK at the transcription level, pink indicates the genes regulated by HNRNPK at the splicing level, purple indicates the genes co-regulated by HNRNPK and NCBP2 at the splicing level, and blue indicates the genes regulated by NCBP2 at the splicing level. Rectangles indicate the target genes of HNRNPK at the transcription level, and ellipse indicates the target gene of HNRNPK and NCBP2 at the splicing level. **(B)** The binding regions of HNRNPK and NCBP2 in splicing target genes, ACTB, AKT1, ASXL1, TCF3, TFRC, and VEGFA. Input the ChIP-seq data of HNRNPK and NCBP2 into the UCSC Genome Browser to obtain the position image of peak in the genome. Purple and red indicate the protein binding sites, and blue indicates the location of genes in the genome. For more detailed information on genes in the network, please refer to Supplementary Table S8.

We selected part of the HNRNPK transcriptional regulation target genes, which are SFs, and the splicing regulation target genes of HNRNPK and NCBP2 to construct the regulatory network of regulation model II (Figure 7A). HNRNPK regulates transcription of NCBP2 and co-regulated splicing of 210 genes with NCBP2. Of the co-regulated genes, 82% (172 out of 210) were found to be associated with the neoplastic process through gene–disease association analysis using the DisGeNET platform (Supplementary Figure S18A). Furthermore, 38% (65 out of 172) of these cancer-related genes are associated with leukemia (Supplementary Figure S18B). Six of them are visualized in Figure 7B, whose RNA-binding positions of HNRNPK and NCBP2 are shown. HNRNPK and NCBP2 bonded mainly to the intron regions of splicing target genes, and the binding sites of two proteins overlap in some regions of several genes. A similar network was also constructed for HNRNPK and SSB (Supplementary Figure S7). These results suggest that DRBP-SFs can link regulatory networks of transcription and AS through regulatory model II.

### Regulatory Model III

A DRBP-SF may also act as a TF in conjunction with another TF regulated by DRBP-SF at the splicing level during transcriptional regulation (Figure 4C).

For example, 35%–40% of the direct transcriptional regulatory targets of HNRNPK, HNRNPL, and TARDBP were also regulated by other TFs, such as scaffold attachment factor B1 (SAFB), which was regulated by these three DRBPs at the splicing level. SAFB binds to the scaffold–matrix attachment region (S–MAR) DNA and forms a molecular assembly point to allow the formation of a “transcriptosomal” complex coupling transcription and RNA processing (Nayler et al., 1998). In contrast, SAFB can interact with HNRNPK, NONO, and TARDBP but not HNRNPL, as shown in their PPI network (Supplementary Figures S13–S17).

**FIGURE 8:**
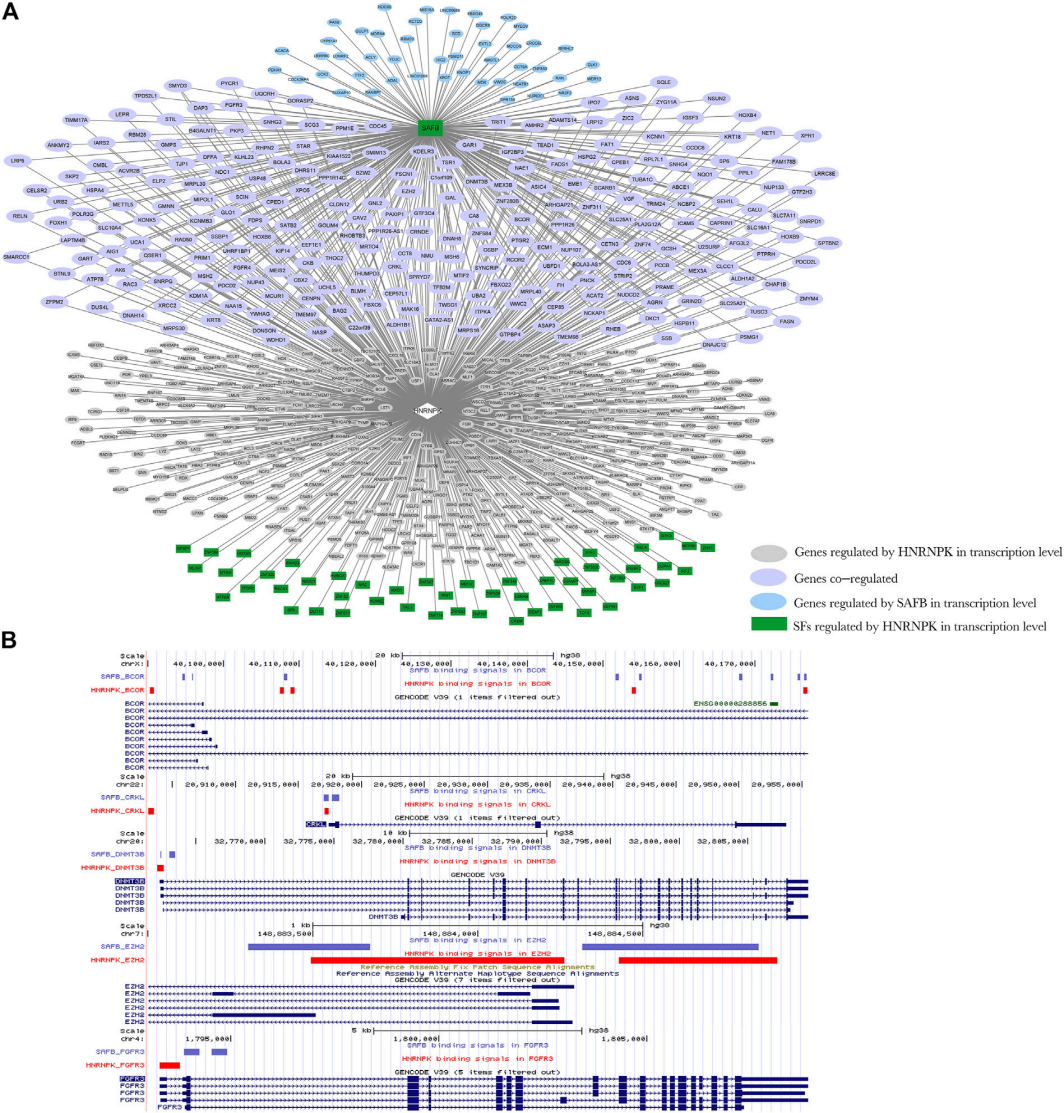
Network diagram of regulation model Ⅲ. **(A)** Heterogeneous nuclear ribonucleoprotein K (HNRNPK) regulates the splicing of scaffold attachment factor B1 (SAFB); HNRNPK and SAFB can jointly regulate the transcription of downstream genes. Green indicates genes that act as transcription factors regulated by HNRNPK at the splicing level, pink indicates genes regulated by HNRNPK at the transcriptional level, blue indicates genes regulated by SAFB at the transcriptional level, and purple indicates genes co-regulated by HNRNPK and nuclear cap–binding protein subunit 2 (NCBP2) at the transcriptional level. Ellipses indicate the target genes of SAFB and HNRNPK at the transcriptional level, and rectangles indicate target genes of HNRNPK at the splicing level. **(B)** The binding regions of HNRNPK and SAFB in transcriptional regulation target genes, BCL6 corepressor, CRK-like proto-oncogene adaptor protein, DNA methyltransferase 3 beta, enhancer of zeste 2 polycomb repressive complex 2 subunit, and fibroblast growth factor receptor 3. Input the ChIP-seq data of HNRNPK and SAFB into the UCSC Genome Browser to obtain the position image of peak in the genome. Purple and red indicate the protein binding sites, and blue indicates the location of genes in the genome. For more detailed information on genes in the network, please refer to Supplementary Table S9.

HNRNPK splicing regulatory target genes, which were TFs, as well as HNRNPK and SAFB transcriptional regulatory target genes, were selected to construct a regulatory network of regulatory model III (Figure 8A). HNRNPK regulated the splicing of SAFB and, together with SAFB, the transcription of 245 genes, 85% (209 out of 245) of which are associated with the neoplastic process (Supplementary Figure S18A). Furthermore, 39% (81 out of 209) of cancer-related genes are associated with leukemia (Supplementary Figure S18B). Five of them are visualized in Figure 8B, whose DNA-binding sites of HNRNPK and SAFB are shown. HNRNPK and SAFB mainly bonded to the promoter regions of their transcriptional regulatory target genes.

### Validation of the Models

To validate the proposed models, we used text mining to search literatures in PubMed using keywords “DRBP-SF & target gene,” “DRBP-SF & transcription–splice,” and “DRBP-SF & co-transcriptional splicing,” where “DRBP-SF” is one of HNRNPK, HNRNPL, NONO, and TARDBP and “target gene” iterates all targets controlled by these four DRBP-SFs in three models connecting both transcriptional and AS regulatory networks. Approximately 600 articles that matched the aforementioned keywords were manually screened and reviewed. However, due to the complexity of biological transcription and post-transcription mechanisms, limitations of the current understanding of DBPs and RBPs, and the lack of technology for DRBP study, these studies mainly focus on PPIs and expression changes of the proteins rather than the transcriptional or AS regulation networks of these DRBP-SFs on their target genes. Nevertheless, we found some evidence that support our models.

For model I, it has been confirmed by many studies that AS is coupled with transcription that permits the sequential recognition of emerging splicing signals by the splicing machinery (Oesterreich et al., 2011). This phenomenon of co-transcriptional splicing is very common in HNRNPs. It has been reported that SET Domain Containing 2 (SETD2) methyltransferase interacts with HNRNPL to control co-transcriptional splicing (Bhattacharya et al., 2021). HNRNPG directly binds to the phosphorylated carboxy terminal domain (CTD) of RNA polymerase II (RNAPII) using RGG motif in its low-complexity region and assembles RNA into large complexes simultaneously. Through interactions with the phosphorylated CTD and nascent RNA, HNRNPG associates co-transcriptionally with RNAPII and regulates AS transcriptome-wide (Zhou et al., 2019). For model II, RBP Sam68 (encoded by KHDRBS1) has previously been identified as a protein partner interacting with androgen receptor (AR) and serves as a co-regulator in AR-dependent transcription and splicing (Stockley et al., 2015). Its transcription is regulated by HNRNPK. Besides, HNRNPK has been shown to indirectly bind RNA by forming a super complex with Sam68. For model III, HNRNPA1 plays a pivotal role in the generation of AR splicing isoforms, such as AR-V7 (Nadiminty et al., 2015), whereas transcription of AR is found to depend on HNRNPK (Capaia et al., 2018). It has also been proven that HNRNPA1 regulates AS through HNRNP particles, a complex composed of multiple HNRNPs (Geuens et al., 2016). These studies confirm that HNRNPK may co-regulate AR splicing through HNRNP particles and HNRNPA1 and act as a partner of AR to co-regulate the downstream transcription process. In summary, although the transcriptional and AS regulatory functions of these DRBP-SFs are often investigated separately in different studies, the evidence of hnRNPs, Sam68 and AR mentioned above supports our three models respectively, in which DRBP-SFs serve as a connection between transcriptional and AS regulations.

## Discussion

In this study, we investigated a class of DRBPs that also functioned as SFs, called DRBP-SFs. These proteins play critical roles in regulating gene expression at both the transcriptional and splicing levels with the capabilities to bind both DNAs and RNAs. By using BETA and SURF to construct regulatory networks in CML, we discovered a two-layer regulatory network system, connecting transcriptional and splicing regulatory networks through DRBP-SFs. Three transcriptional and splicing co-regulatory models were proposed by investigating the two-layer regulatory network system controlled by four DRBP-SFs, namely, HNRNPK, HNRNPL, NONO, and TARDBP. In model I, there are some genes directly regulated at the transcriptional and splicing levels by the same DRBP-SFs that function as TFs and SFs simultaneously, which might be involved in co-transcriptional splicing for rapid expression. In models II and III, DRBP-SFs dually control transcriptional and splicing networks through direct and indirect mechanisms, respectively, in which they collaborate with their own targets at one regulatory level and regulate other targets at the other regulatory level. Our results provide supporting evidence for understanding the dual role of DRBP-SFs in transcriptional control and AS.

Through motif analysis, we further explored how HNRNPK binds to its two-level co-regulated genes in model I and found that HNRNPK directly binds to its promoters in most cases while indirectly binding to its pre-mRNA through other SFs in most cases; moreover, in a small number of cases, HNRNPK directly binds to its pre-mRNA targets. Further, we speculate that in regulatory model I, HNRNPK regulates transcription and splicing in a synergistic rather than a competitive manner. Because HNRNPK has multiple DNA and RNA binding domains, DNA and RNA are possible to bind HNRNPK simultaneously (Supplementary Table S5). This co-binding allows HNRNPK to regulate both transcription and splicing at the same time, which is called co-transcriptional splicing. As co-transcriptional splicing often occurs in fast transcription (Naftelberg et al., 2015), our study also supports that the target genes of HNRNPK in regulatory model I are highly expressed in CML, because rapid transcription is more likely to produce highly expressed genes. However, further study would be needed to confirm this hypothesis.

The four DRBP-SFs have been reported to play vital roles in cancers and other important BPs. HNRNPK regulates a wide range of BPs and disease pathogenesis, which is central to many cellular events, including long noncoding RNA (lncRNA) regulation, cancer development, and bone homeostasis (Wang et al., 2020). HNRNPL directly regulates the AS of various RNAs, including those encoding the AR as well as the key lineage-specific prostate cancer oncogene (Fei et al., 2017). NONO, a multifunctional nuclear protein rarely functioning alone, has been found to cause many types of cancer (Feng, Li et al., 2020). Mutations in TARDBP caused familial amyotrophic lateral sclerosis (ALS) and frontotemporal dementia (FTD) (Feng et al., 2020; Klim et al., 2021). Genes in the dual network regulated by HNRNPK in CML, namely, proteasome activator subunit 2 (PRAME) (Oehler et al., 2009), enhancer of zeste 1 polycomb repressive complex 2 subunit (EZH1) (Xie et al., 2016) from model I, AKT serine–threonine kinase 1 (AKT1) (Butt et al., 2020), ASXL transcriptional regulator 1 (ASXL1) (Tran and Wong 2021), transcription factor 3 (TCF3) (Kesy and Januszkiewicz-Lewandowska 2015), and vascular endothelial growth factor A (VEGFA) (Lakkireddy et al., 2016) from model II, BCL6 corepressor (BCOR) (Sportoletti et al., 2021), CRK-like proto-oncogene adaptor protein (CRKL) (Nichols et al., 1994), DNA methyltransferase 3 beta (DNMT3B) (Mizuno et al., 2001), enhancer of zeste 2 polycomb repressive complex 2 subunit (EZH2) (Xie et al., 2016), and fibroblast growth factor receptor 3 (FGFR3) (Dvorakova et al., 2001) from model Ⅲ, have been reported to be closely related to the occurrence and development of CML (Stelzer et al., 2016). Overall, a high proportion of co-regulated genes in the three regulatory models of HNRNPK are associated with neoplastic process, some of which are associated with leukemia (Supplementary Tables S6, S7; Supplementary Figure S18), indicating that HNRNPK is a key factor in CML. Furthermore, to a certain extent, our proposed models reveal the function mechanism of HNRNPK in CML. As DRBP-SFs are key players in transcriptional and post-transcriptional events, these observations add to a growing body of evidence indicating that DRBP-SFs may promote cancer development after a key oncogenic event by altering various cancer-associated downstream targets through the establishment of highly intricate regulatory networks, thus amplifying the phenotypic consequences of the initial transforming hit(s) through a “ripple effect” (Pereira et al., 2017). In this scenario, DRBP-SFs act mainly as amplifiers of oncogenic driver mutations.

This study has several limitations. The DNA- and RNA-binding data of DRBP-SFs are from different sources, and ChIP-seq and eCLIP experiments were performed by different laboratories. Furthermore, we still lack an experimental technique that can investigate how DRBP-SFs bind to DNA and RNA at the same time. Due to the limitations of the current technologies and followed bioinformatics analysis methods, the transcription and splicing networks may not be able to truly, accurately, and completely reflect the actual situation in cells. The three regulatory models are worthy of validation in more cells and DRBP-SFs. Besides, although we observed that HNRNPK binds to DNA directly and to RNA indirectly on many target genes in regulatory model I, it requires more interaction data to support the finding. For regulatory models II and III, whether the DRBP-SFs and the coordinated and regulated TFs–SFs possess direct physical interaction would also require further experimental verification.

In conclusion, DRBP-SFs are key players in transcriptional and post-transcriptional events. The combination of versatility of their DNA- and RNA-binding domains and their structural flexibility enables DRBP-SFs to control the metabolism of a large array of transcripts. The DRBP-SF regulatory networks we have constructed here suggested a novel two-layer regulatory system on both transcriptional and splicing levels where DRBP-SFs are demonstrated to co-regulate DNA and RNA in conjunction. For this, three regulatory models were proposed with supporting evidence. This study can provide new ideas for further mechanistic research on DRBP-SFs and their two-layer gene regulatory systems that may play critical roles in cancer.

## Data Availability

The original contributions presented in the study are included in the article/Supplementary Material; further inquiries can be directed to the corresponding authors.

## Glossary

DRBPs: DNA- and RNA-binding proteins
ChIP-seq: Chromatin immunoprecipitation
CLIP-seq: Crosslinking immunoprecipitation
eCLIP: Enhanced CLIP
BETA: Binding and expression target analysis
NBPs: Nucleic acid–binding proteins
DBPs: DNA-binding proteins
RBPs: RNA-binding proteins
TFs: Transcription factors
SFs: Splicing factors
GO: Gene Ontology
BP: Biological process
CC: Cellular component
MF: Molecular function
KEGG: Kyoto Encyclopedia of Genes and Genomes
CML: Chronic myeloid leukemia
GEPIA: Gene expression profiling interactive analysis
AS: Alternative splicing
SE: Exon skipping
A3SS: Alternative 3′splicing
A5SS: Alternative 5′splicing
RI: Intron retention
PPIs: Protein–protein interactions
lncRNA: Long noncoding RNA
ALS: Amyotrophic lateral sclerosis
FTD: Frontotemporal dementia
HNRNPK: Heterogeneous nuclear ribonucleoprotein K
HNRNPL: Heterogeneous nuclear ribonucleoprotein L
NONO: Non-POU domain–containing octamer–binding protein
TARDBP: TAR DNA-binding protein 43
UCA1: Urothelial cancer associated 1
TFE2: Transcription factor E2-α
ALDH1A2: Aldehyde dehydrogenase family 1
ARAP1: ArfGAP with RhoGAP domain, ankyrin repeat, and PH domain 1
CPED1: Cadherin-like and PC-esterase domain–containing 1
DHRS11: Dehydrogenase/reductase 11
HMGCS1: 3-hydroxy-3-methylglutaryl-CoA synthase 1
SMYD3: SET and MYND domain–containing 3
SRSF2: Serine/arginine-rich splicing factor 2
NCBP2: Nuclear cap–binding protein subunit 2
SSB: Small RNA-binding exonuclease protection factor La
CBC: Cap-binding complex
PSME2: Proteasome activator subunit 2
SAFB: Scaffold attachment factor B
SETD2: SET domain–containing 2
CTD: Carboxy terminal domain
RNAPII: RNA polymerase II
AR: Androgen receptor
PRAME: PRAME nuclear receptor transcriptional regulator
EZH1: Enhancer of zeste 1 polycomb repressive complex 2 subunit
AKT1: AKT serine–threonine kinase 1
ASXL1: ASXL transcriptional regulator 1
TCF3: Transcription factor 3
VEGFA: Vascular endothelial growth factor A
BCOR: BCL6 corepressor
CRKL: CRK-like proto-oncogene, adaptor protein
DNMT3B: DNA methyltransferase 3 beta
EZH2: Enhancer of zeste 2 polycomb repressive complex 2 subunit
FGFR3: Fibroblast growth factor receptor 3
