# Supplementary material for: DNA- and RNA-Binding Proteins Linked Transcriptional Control and Alternative Splicing Together in a Two-Layer Regulatory Network System of Chronic Myeloid Leukemia
Source: Front Mol Biosci. 2022 Aug 16;9:920492. doi: 10.3389/fmolb.2022.920492 (PMC9425088; doi:10.3389/fmolb.2022.920492)
Supplement: Supplementary file 1 [file DataSheet2.DOCX]

Supplementary Material

# Supplementary Figures and Tables

## Supplementary Figures


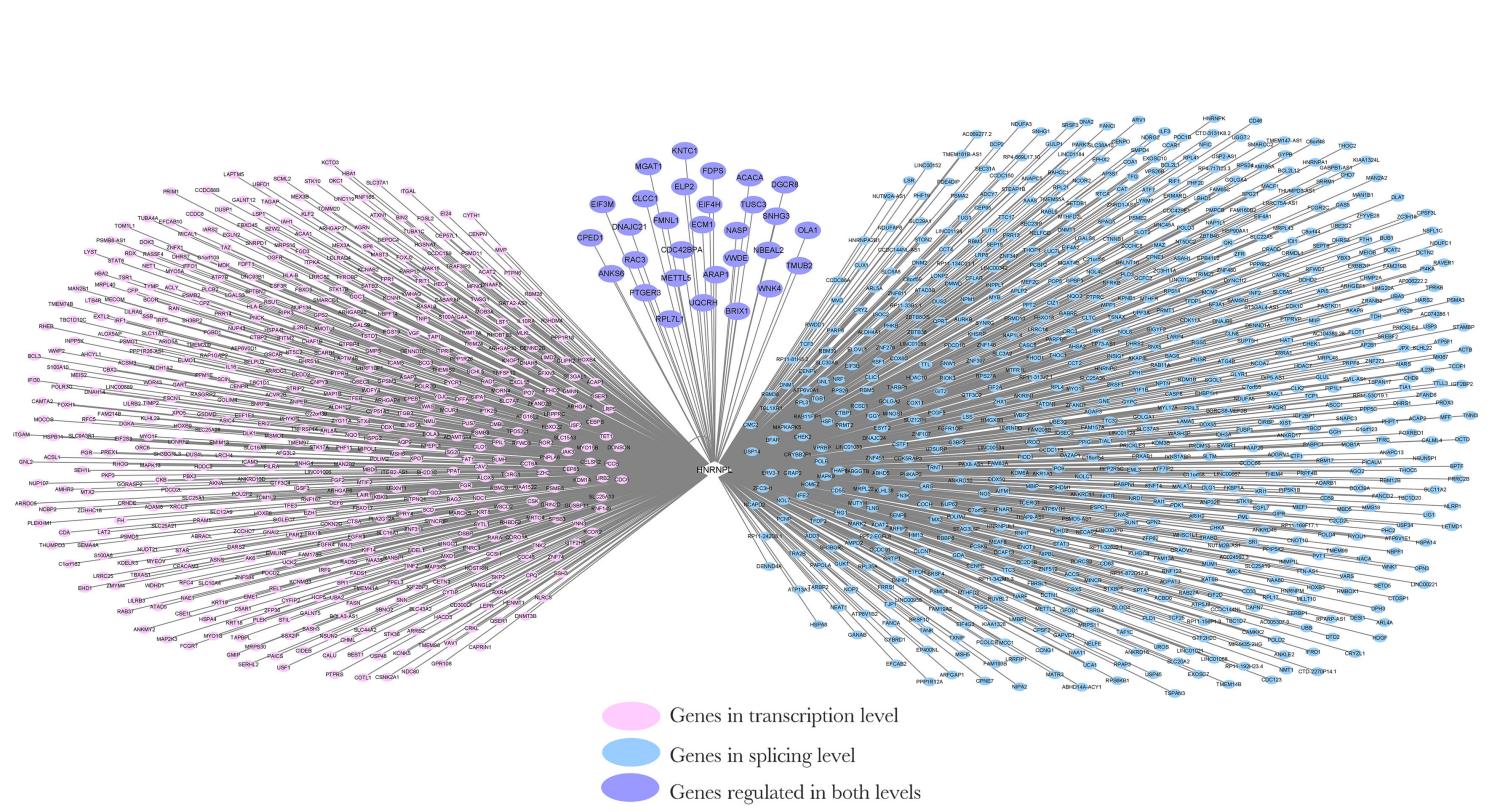


Figure S1. Two-layer regulatory network of HNRNPL. Pink are the target genes of transcriptional regulatory network of HNRNPL, blue are the target genes of splicing regulatory network of HNRNPL, purple are the co-regulated of HNRNPL in transcriptional and splicing regulatory level.For more detailed information on genes in the network, please refer to the Table S7.


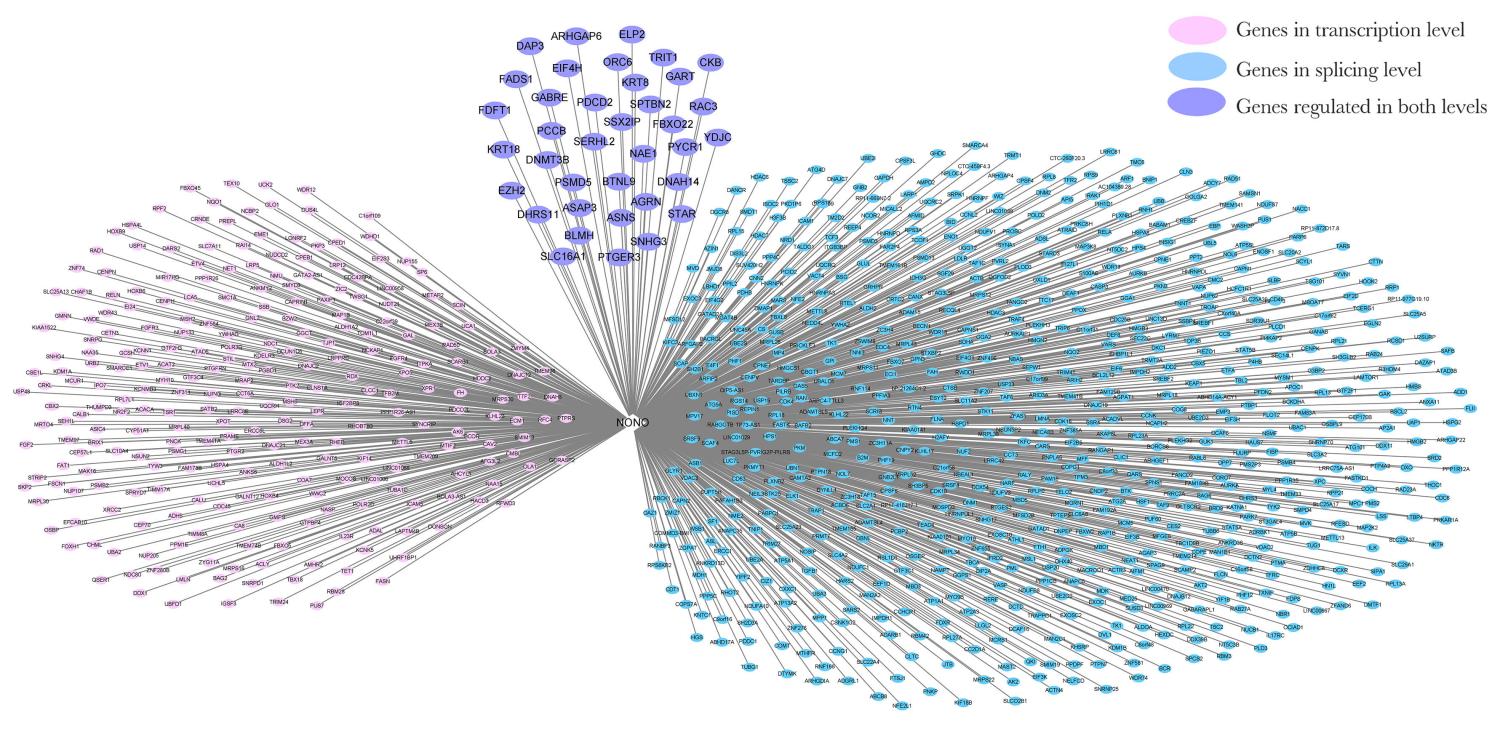


Figure S2. Two-layer regulatory network of NONO. Pink are the target genes of transcriptional regulatory network of NONO, blue are the target genes of splicing regulatory network of NONO, purple are the co-regulated of NONO in transcriptional and splicing regulatory level.For more detailed information on genes in the network, please refer to the Table S7.


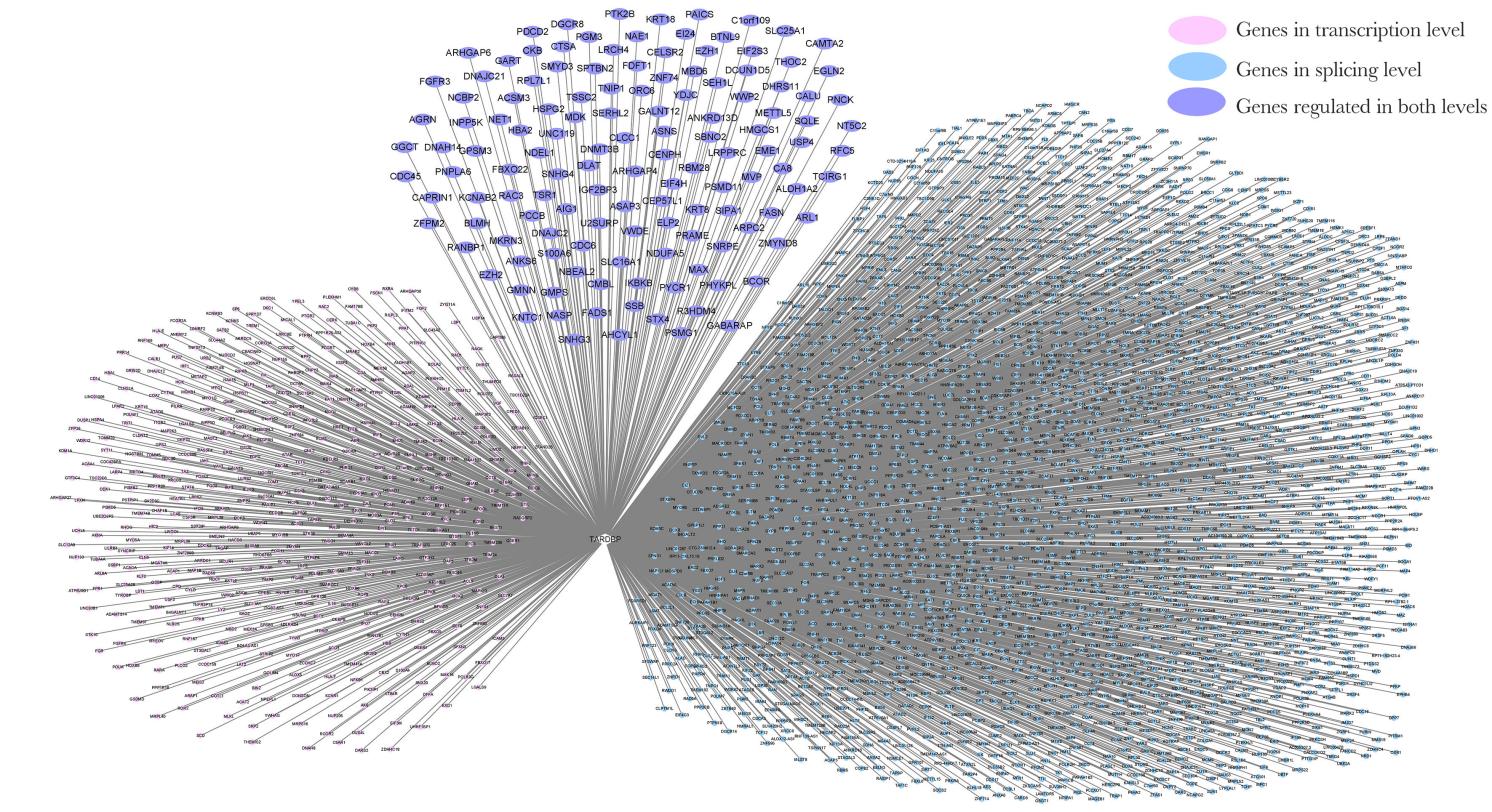


Figure S3. Two-layer regulatory network of TARDBP. Pink are the target genes of transcriptional regulatory network of TARDBP, blue are the target genes of splicing regulatory network of TARDBP, purple are the co-regulated of TARDBP in transcriptional and splicing regulatory level.For more detailed information on genes in the network, please refer to the Table S7.


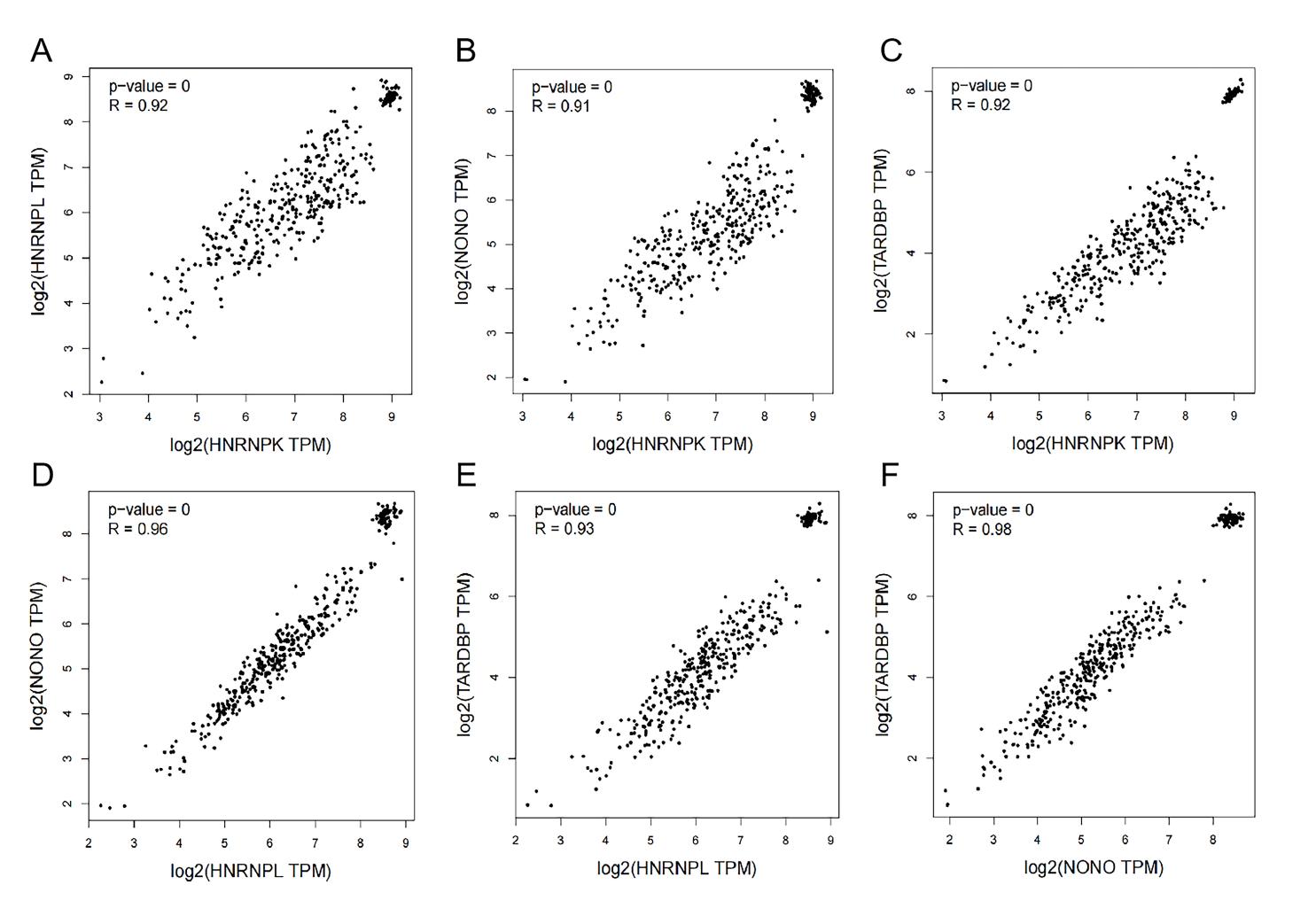


Figure S4. Gene correlations between HNRNPK, HNRNPL, NONO, and TARDBP in Chronic Myeloid Leukemia.


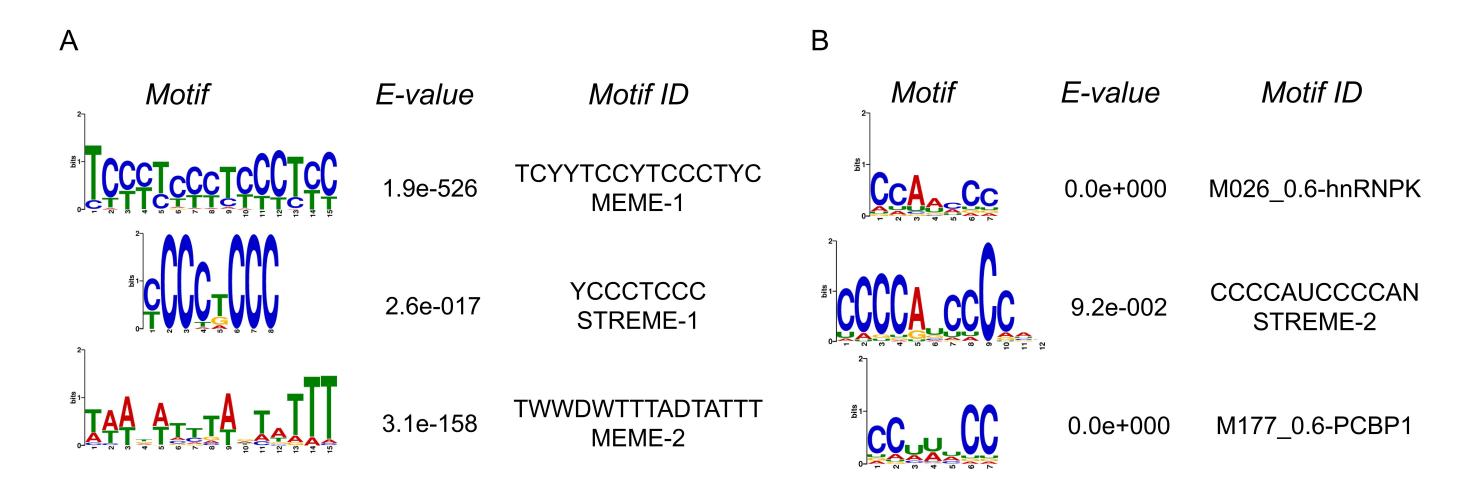


Figure S5**.** The DNA binding motifs and RNA binding motifs of HNRNPK. (A) Three DNA binding motifs of HNRNPK were analyzed from ChIP-seq data. (B) Three RNA binding motifs of HNRNPK were analyzed from CLIP-seq data.


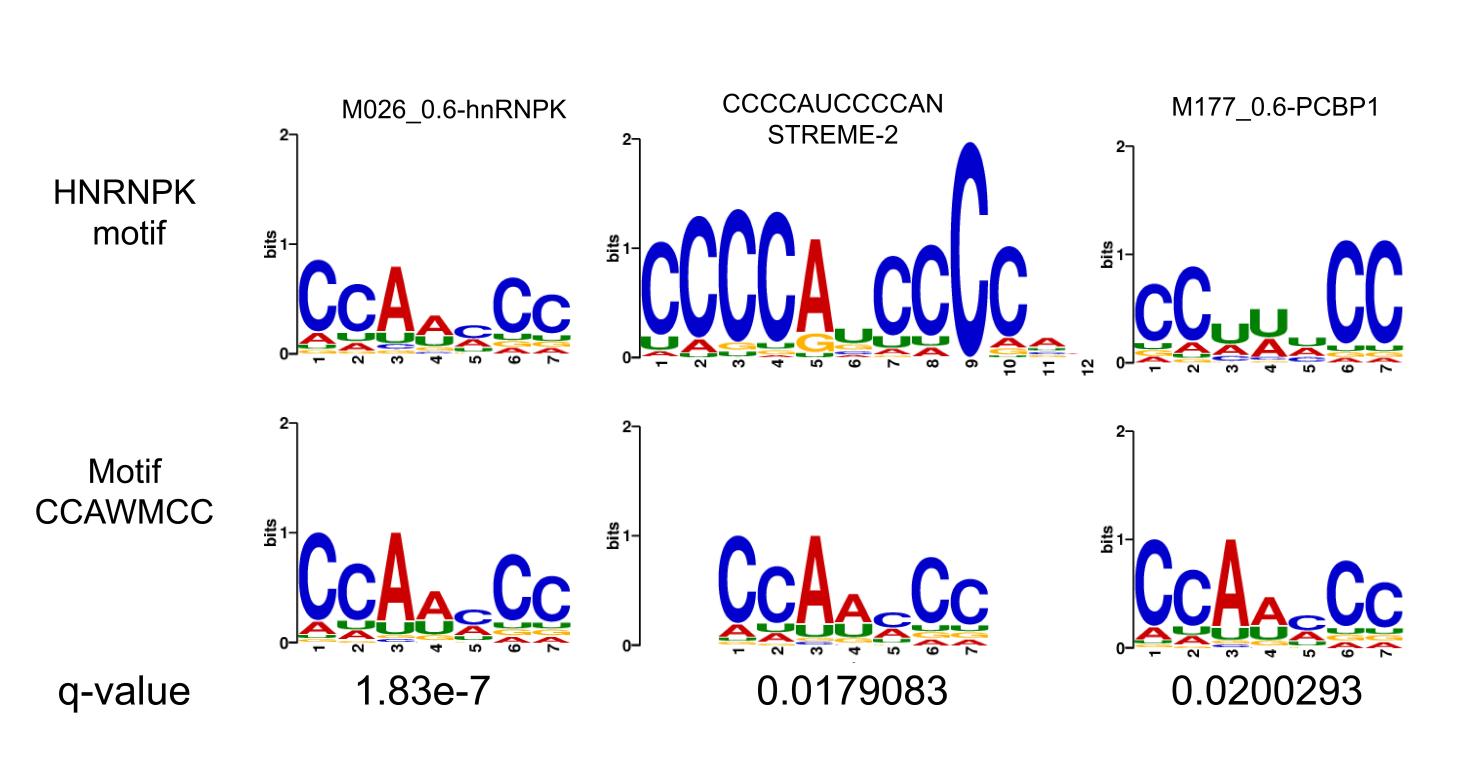


Figure S6. The three motifs most similar with CCAWMCC motifs which is an *in vitro* RNA direct binding motif from CISBP-RNA database with q-value < 0.05 from 87 RNA binding motifs of HNRNPK from MEME-chip.


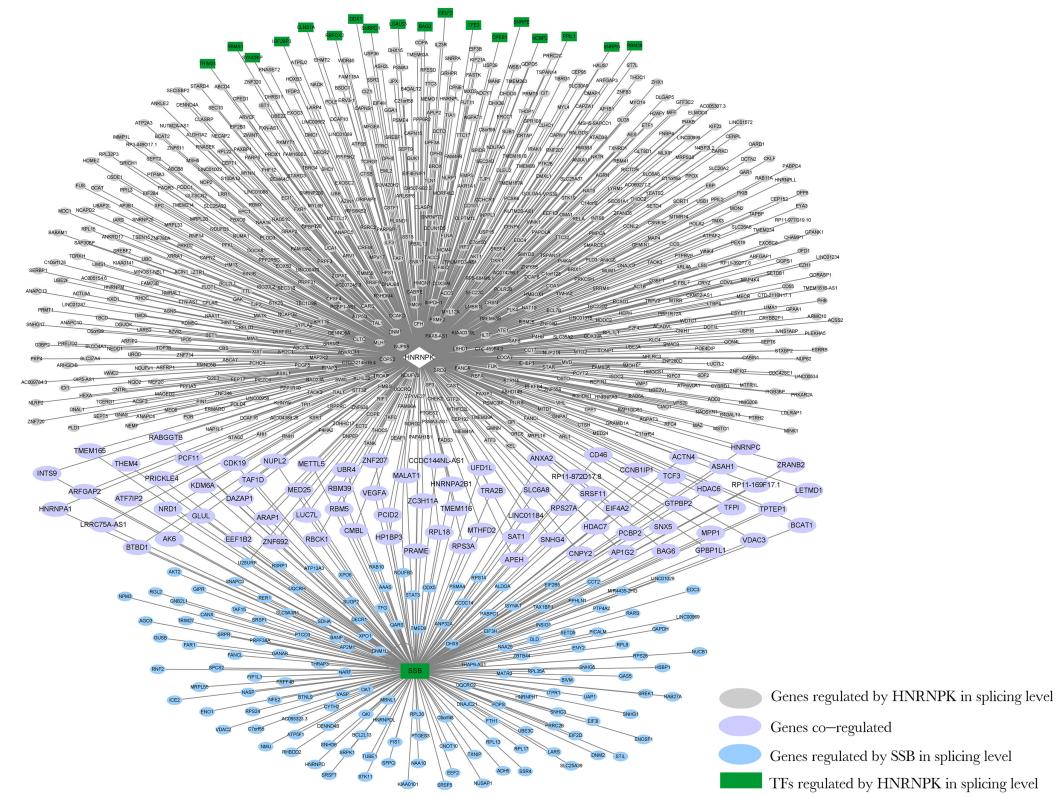


Figure S7. Network diagram of the regulatory model Ⅱ. HNRNPK regulates the transcription of SSB, and then HNRNPK and SSB can jointly regulate the splicing of genes. Green is a gene regulated by HNRNPK in transcription level, gray is a gene regulated by HNRNPK in splicing level, purple is a gene co-regulated by HNRNPK and SSB in splicing level, and blue is a gene regulated by SSB in splicing level. Rectangle is a target gene in transcription level, and ellipse is a target gene in splicing level.For more detailed information on genes in the network, please refer to the Table S8.


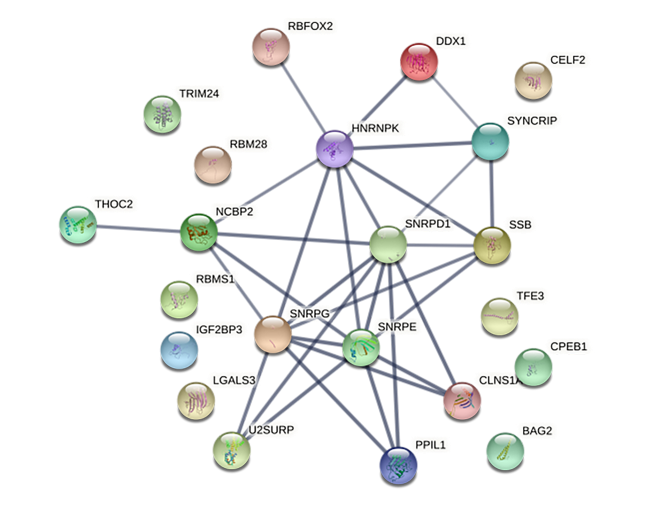


Figure S8. Protein-protein interaction (PPI) analysis of HNRNPK and the SFs in the target genes of transcriptional regulatory network of HNRNPK for regulatory model Ⅱ. Large sizes and dark colors of edges meant high value of combined scores. High confidence score of 0.7 was selected to construction PPI network. There are PPIs between HNRNPK and NCBP2, HNRNPK and SSB.


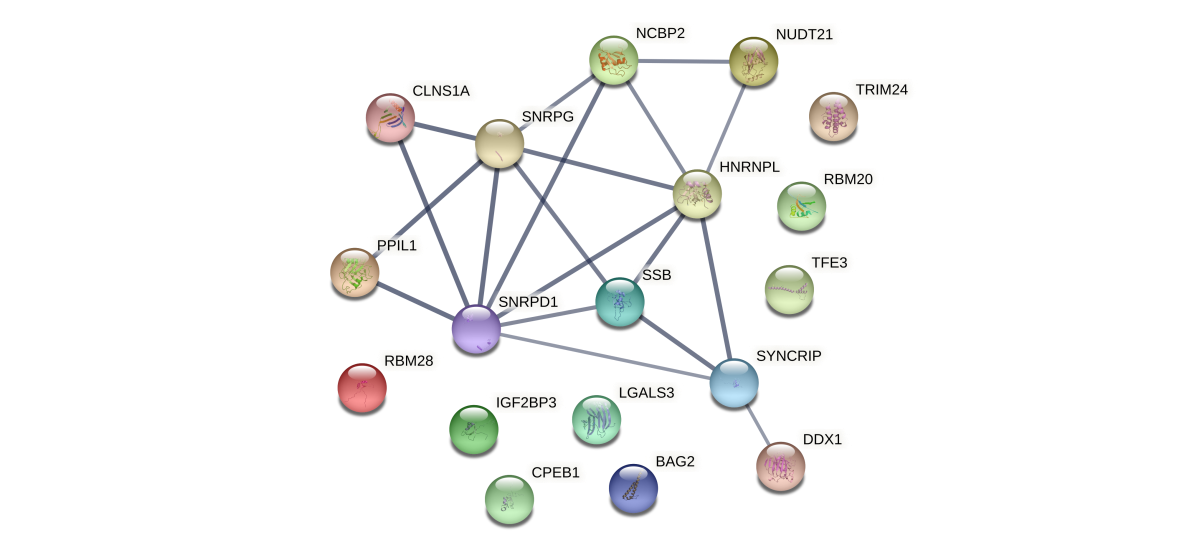


Figure S9. Protein-protein interaction (PPI) analysis of HNRNPL and the SFs in the target genes of transcriptional regulatory network of HNRNPL for regulatory model Ⅱ. Large sizes and dark colors of edges meant high value of combined scores. High confidence score of 0.7 was selected to construction PPI network. There are PPIs between HNRNPL and NCBP2, HNRNPL and SSB.


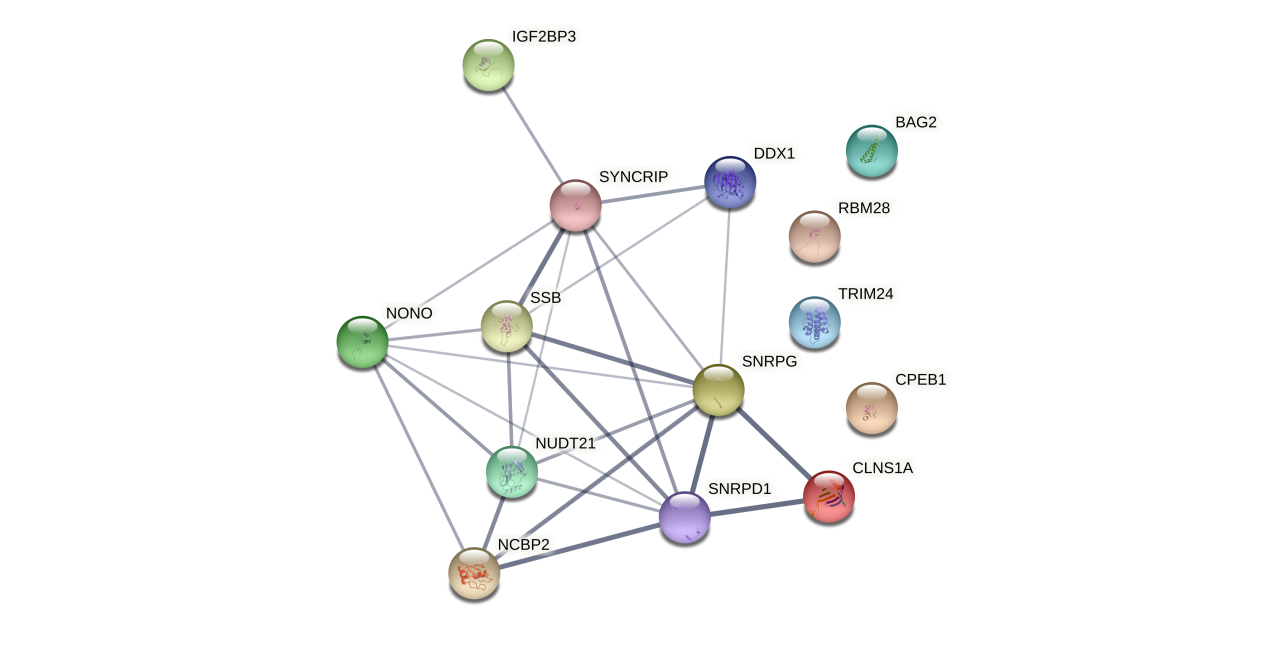


Figure S10. Protein-protein interaction (PPI) analysis of NONO and the SFs in the target genes of transcriptional regulatory network of NONO for regulatory model Ⅱ. Large sizes and dark colors of edges meant high value of combined scores. Medium confidence score of 0.4 was selected to construction PPI network. There are PPIs between NONO and NCBP2, NONO and SSB.


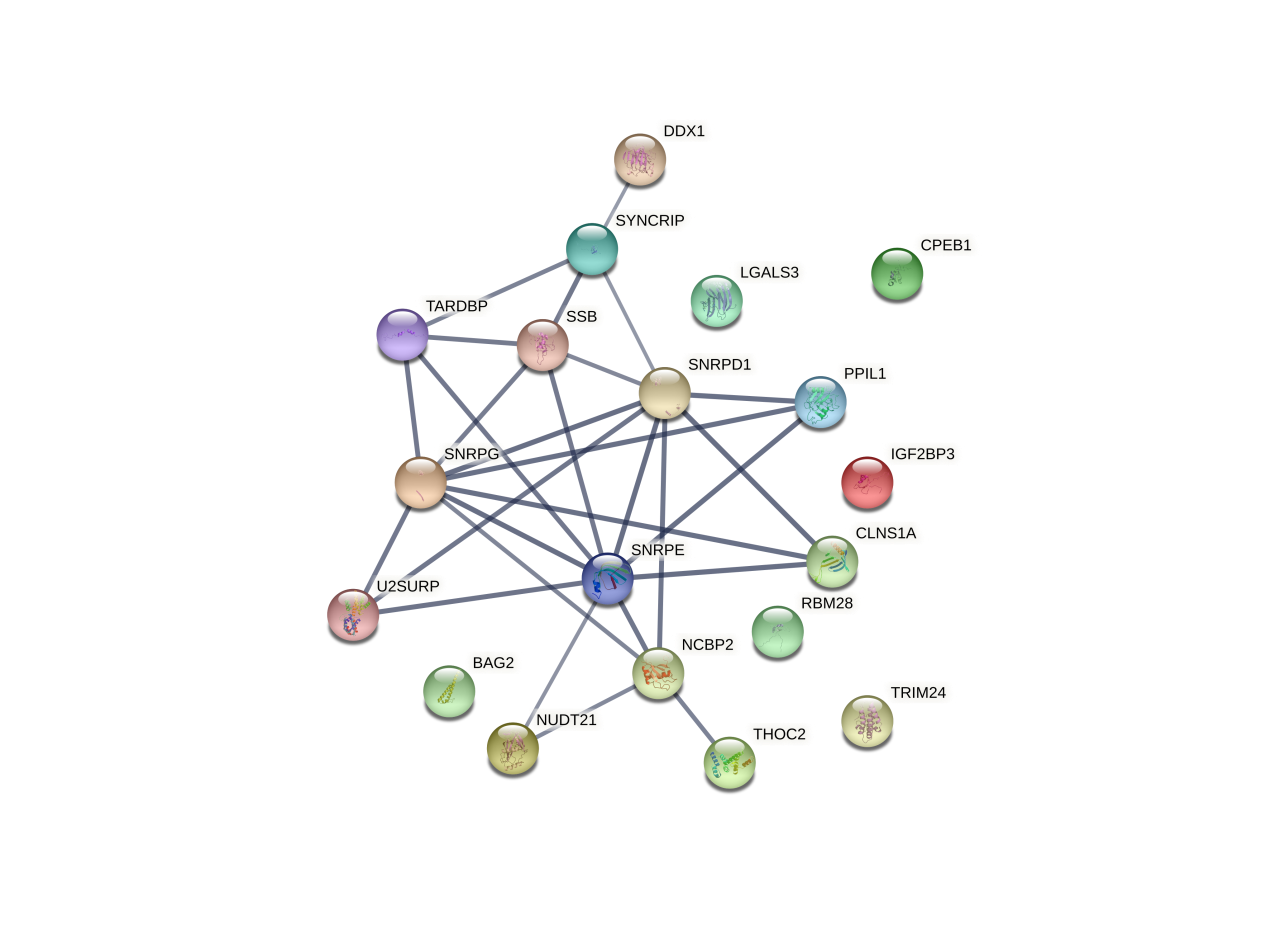


Figure S11. Protein-protein interaction (PPI) analysis of TARDBP and the SFs in the target genes of transcriptional regulatory network of TARDBP for regulatory model Ⅱ. Large sizes and dark colors of edges meant high value of combined scores. High confidence score of 0.7 was selected to construction PPI network. There is PPI in TARDBP and SSB, but no PPI between TARDBP and NCBP2.


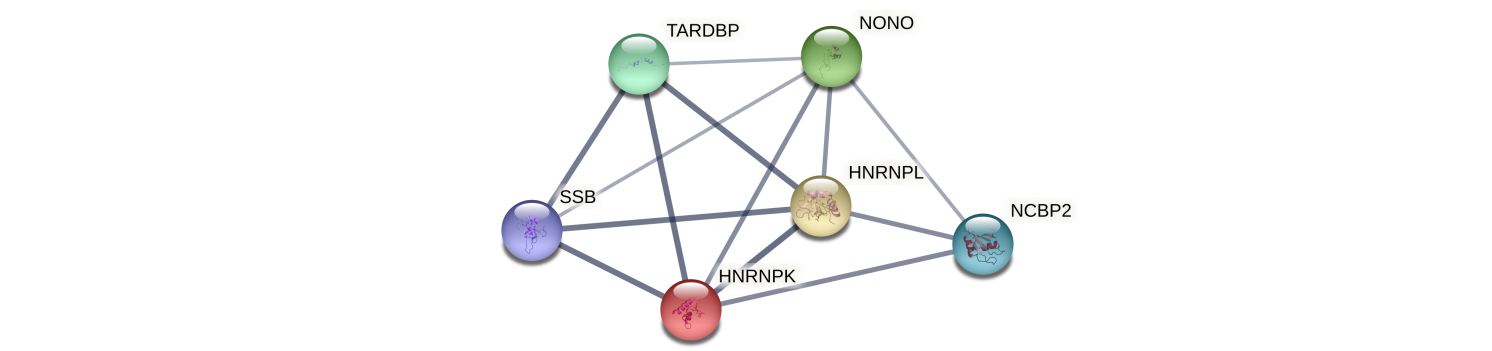


Figure S12. Protein-protein interaction (PPI) analysis of HNRNPK, HNRNPL, NONO, TARDBP, NONO and NCBP2 for regulatory model Ⅱ. Large sizes and dark colors of edges meant high value of combined scores. Medium confidence score of 0.4 was selected to construction PPI network.


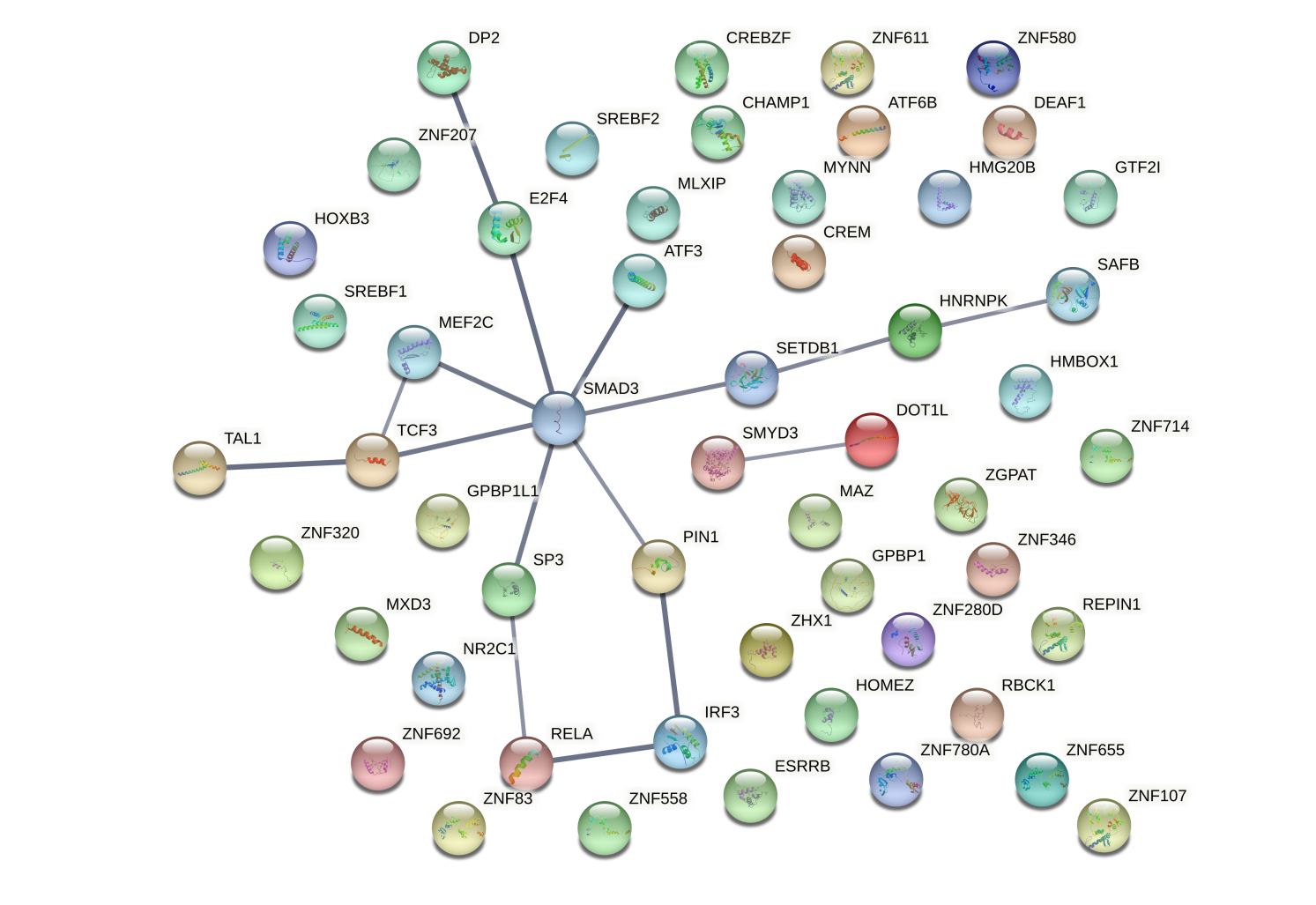


Figure S13. Protein-protein interaction (PPI) analysis of HNRNPK and the TFs in the target genes of splicing regulatory network of HNRNPK for regulatory model Ⅲ. Large sizes and dark colors of edges meant high value of combined scores. High confidence score of 0.7 was selected to construction PPI network. There is PPI between HNRNPK and SAFB.


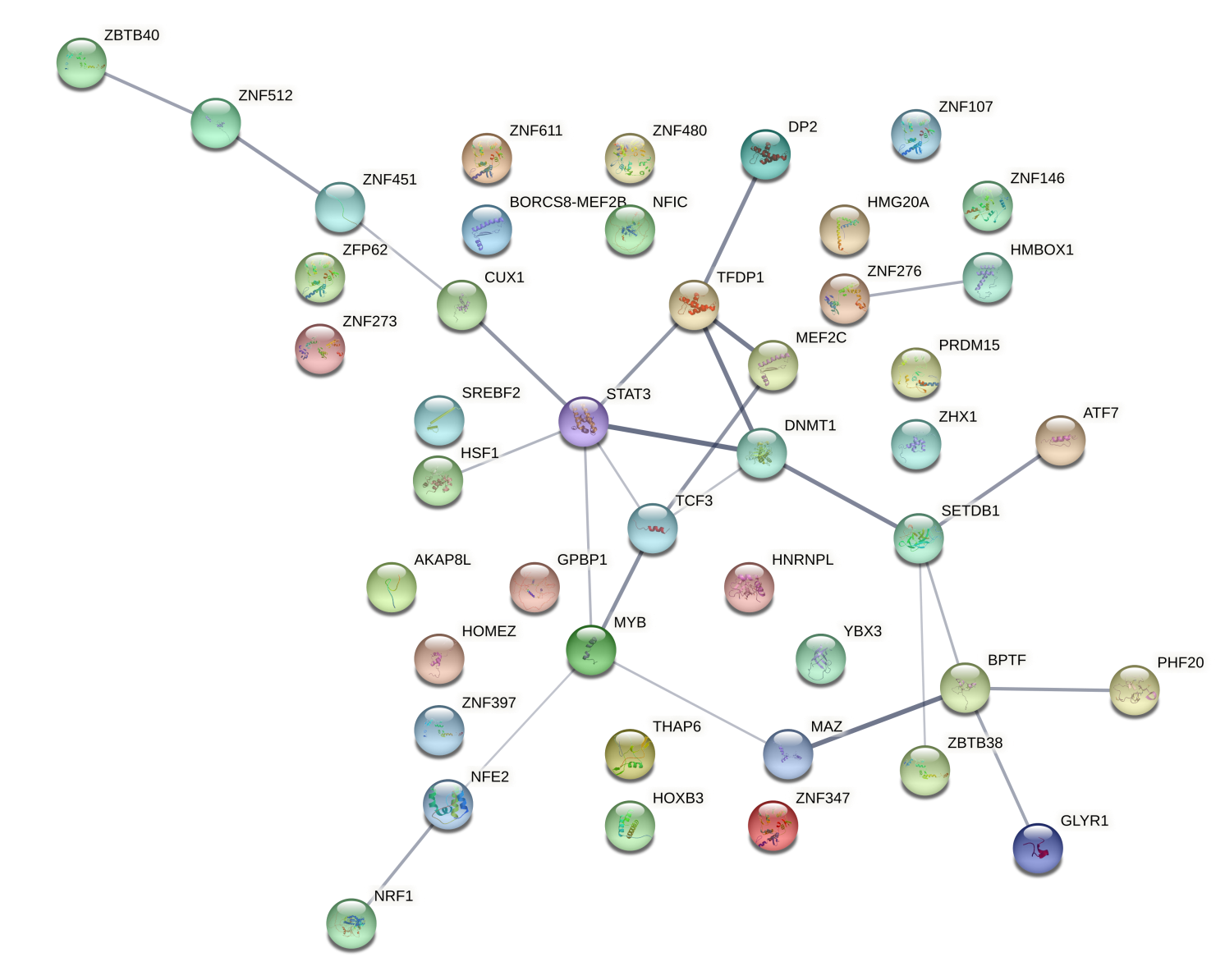


Figure S14. Protein-protein interaction (PPI) analysis of HNRNPL and the TFs in the target genes of splicing regulatory network of HNRNPL for regulatory model Ⅲ. Large sizes and dark colors of edges meant high value of combined scores. Medium confidence score of 0.4 was selected to construction PPI network. There is no PPI between HNRNPL and the TFs in the target genes of splicing regulatory network of HNRNPL.


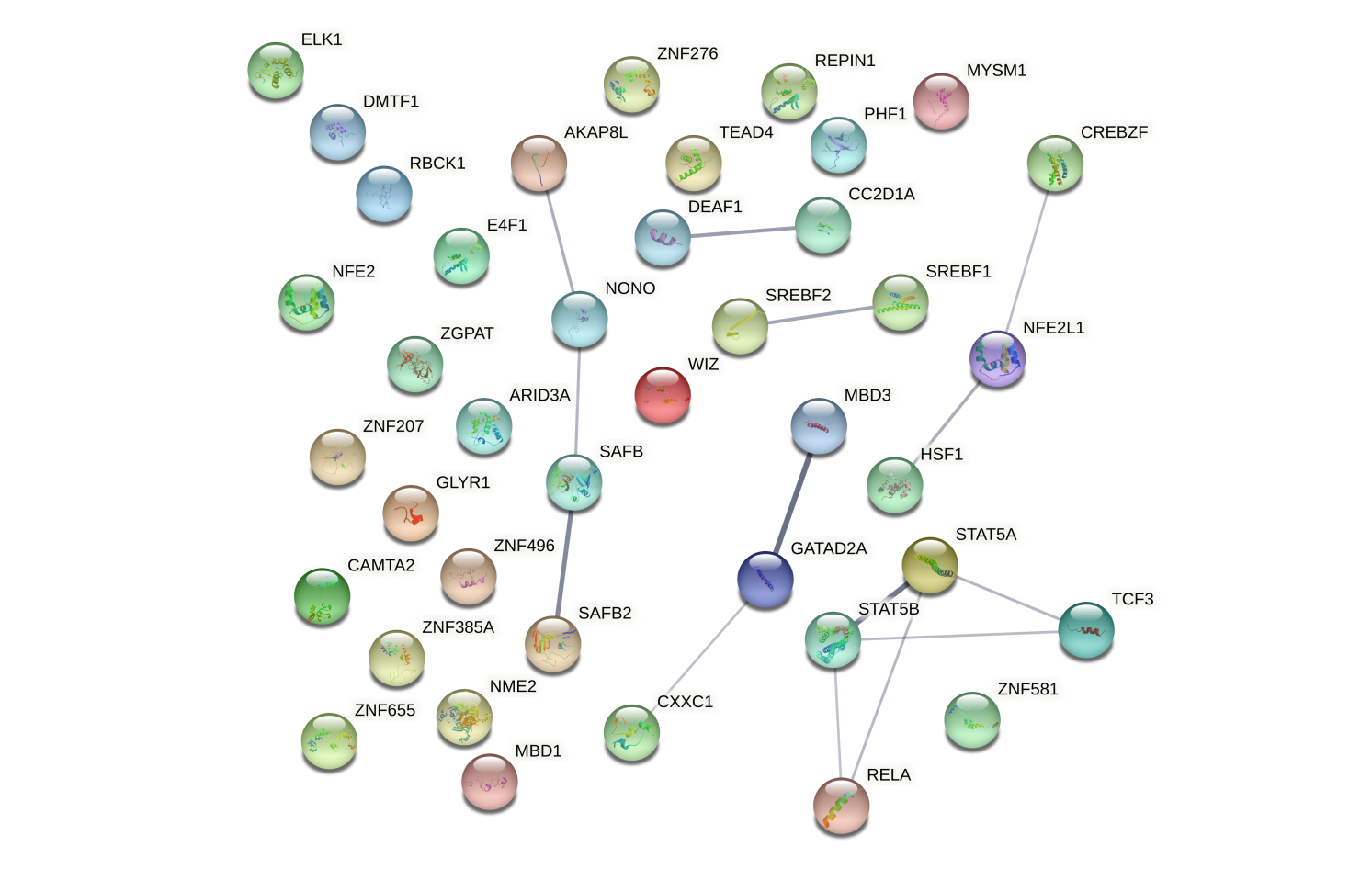


Figure S15. Protein-protein interaction (PPI) analysis of NONO and the TFs in the target genes of splicing regulatory network of NONO for regulatory model Ⅲ. Large sizes and dark colors of edges meant high value of combined scores. Medium confidence score of 0.4 was selected to construction PPI network. There is a PPI between NONO and SAFB.


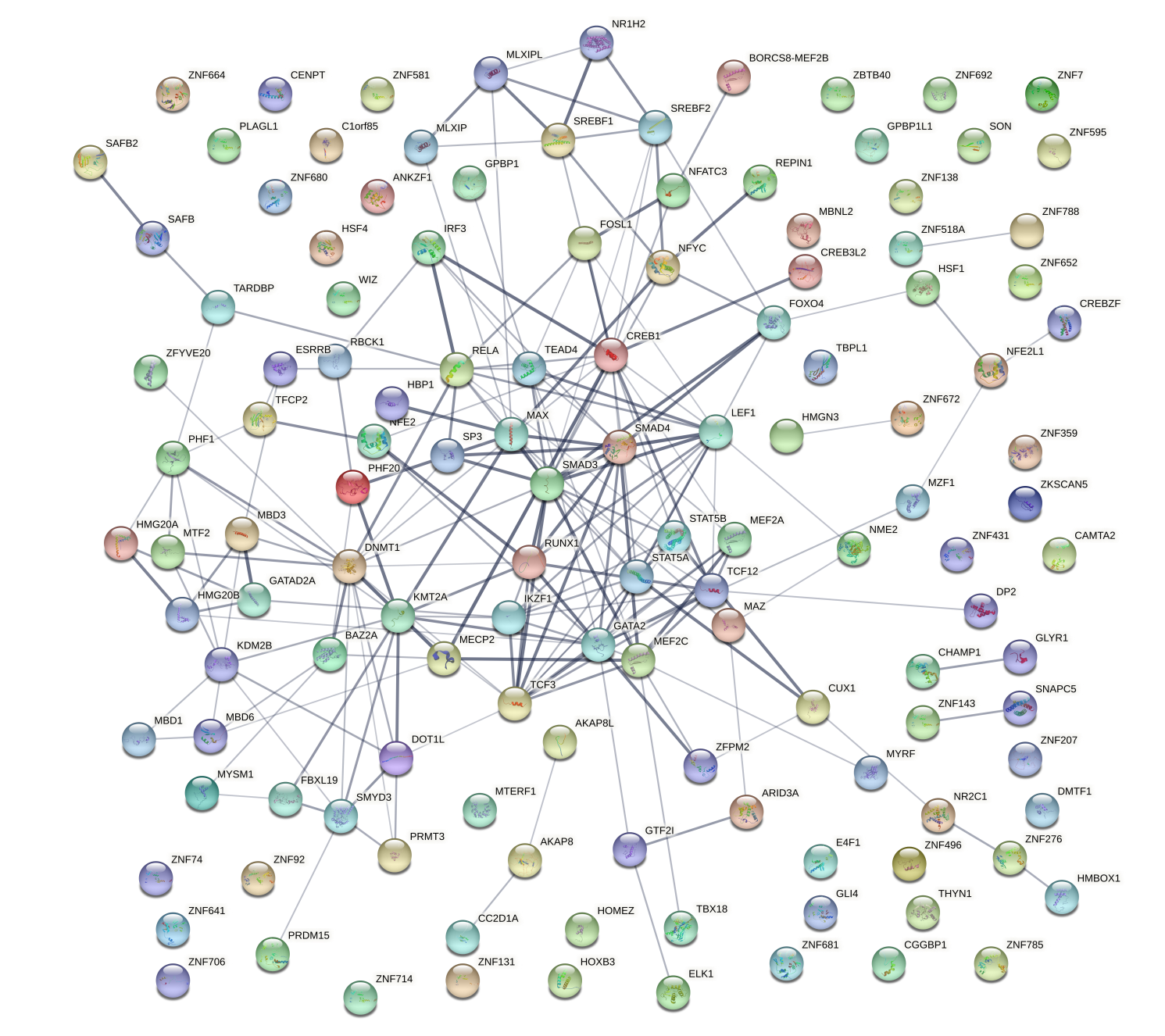


Figure S16. Protein-protein interaction (PPI) analysis of TARDBP and the TFs in the target genes of splicing regulatory network of TARDBP for regulatory model Ⅲ. Large sizes and dark colors of edges meant high value of combined scores. Medium confidence score of 0.4 was selected to construction PPI network. There is a PPI between TARDBP and SAFB.


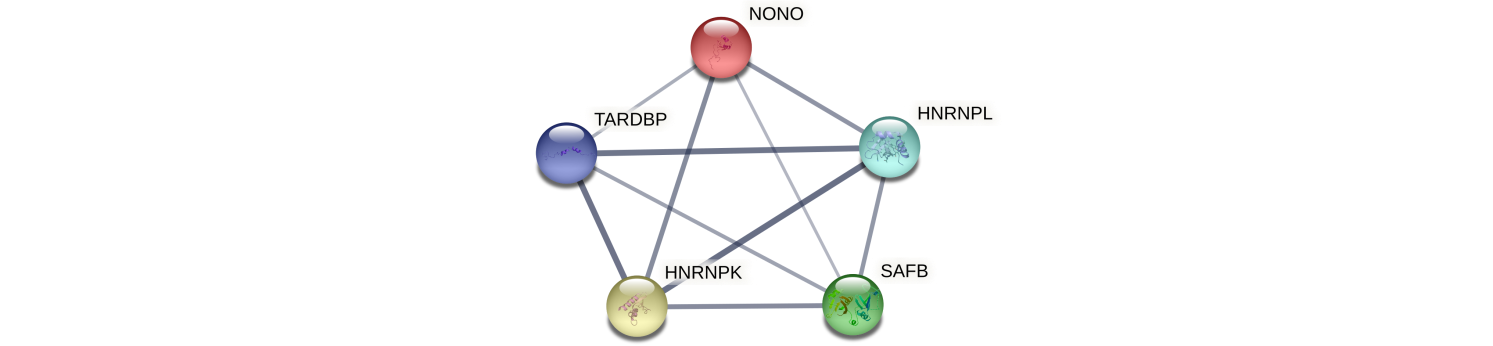


Figure S17. Protein-protein interaction (PPI) analysis of HNRNPK, HNRNPL, NONO, TARDBP and SAFB for regulatory model Ⅲ. Large sizes and dark colors of edges meant high value of combined scores. Medium confidence score of 0.4 was selected to construction PPI network.


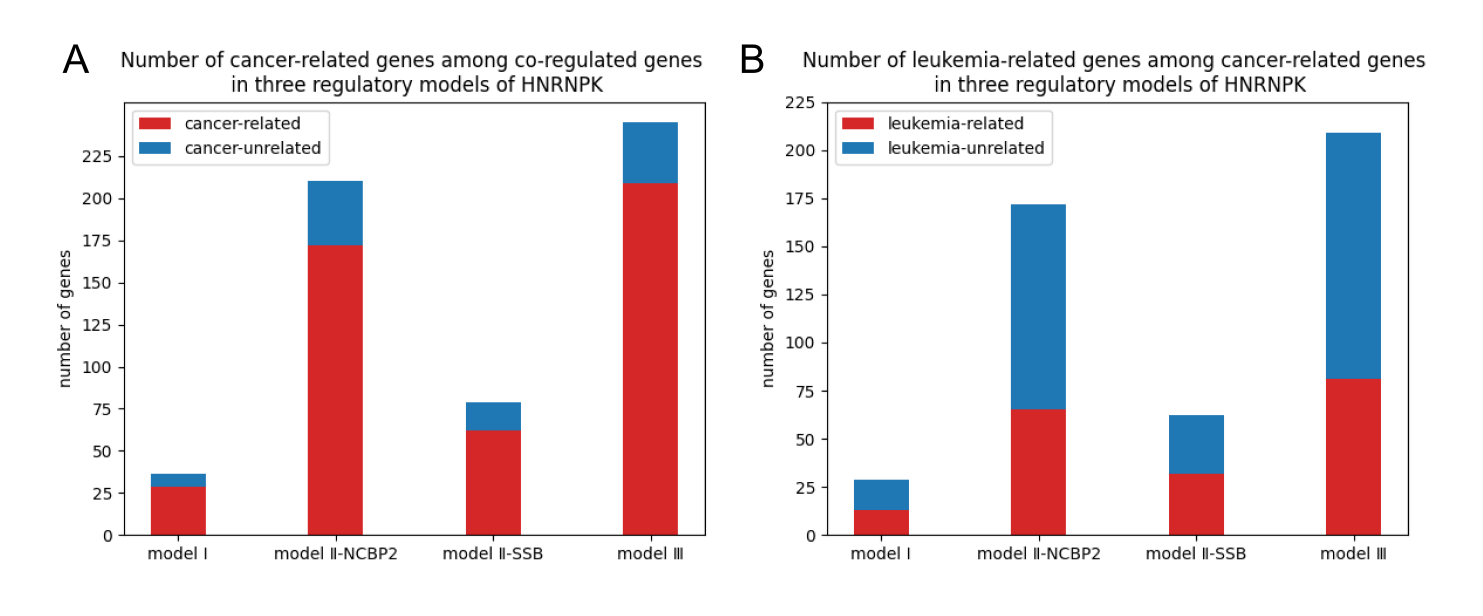


Figure S18. Statistical results of gene-disease association analysis with co-regulated genes in three regulatory models of HNRNPK using DisGeNET platform. (A) Number of cancer-related genes among co-regulated genes in three regulatory models of HNRNPK. (B) Number of leukemia-related genes among cancer-related genes in three regulatory models of HNRNPK.

## Supplementary Table

Table S7. Statistical data of gene-disease association analysis with co-regulated genes in three regulatory models of HNRNPK using DisGeNET platform

|  | **Model I** | **ModelⅡ-NCBP2** | **Model Ⅱ-SSB** | **Model Ⅲ** |
| --- | --- | --- | --- | --- |
| Co-regulated genes account | 36 | 210 | 79 | 245 |
| Cancer-related genes account | 29 | 172 | 62 | 209 |
| Cancer-related genes account for the proportion of co-regulated genes | 81% | 82% | 78% | 85% |
| leukemia-related genes account | 13 | 65 | 32 | 81 |
| leukemia-related genes account for the proportion of Cancer-related genes | 45% | 38% | 52% | 39% |
